# [^18^F]F-DED PET imaging of reactive astrogliosis in neurodegenerative diseases: preclinical proof of concept and first-in-human data

**DOI:** 10.1186/s12974-023-02749-2

**Published:** 2023-03-11

**Authors:** Anna Ballweg, Carolin Klaus, Letizia Vogler, Sabrina Katzdobler, Karin Wind, Artem Zatcepin, Sibylle I. Ziegler, Birkan Secgin, Florian Eckenweber, Bernd Bohr, Alexander Bernhardt, Urban Fietzek, Boris-Stephan Rauchmann, Sophia Stoecklein, Stefanie Quach, Leonie Beyer, Maximilian Scheifele, Marcel Simmet, Emanuel Joseph, Simon Lindner, Isabella Berg, Norman Koglin, Andre Mueller, Andrew W. Stephens, Peter Bartenstein, Joerg C. Tonn, Nathalie L. Albert, Tania Kümpfel, Martin Kerschensteiner, Robert Perneczky, Johannes Levin, Lars Paeger, Jochen Herms, Matthias Brendel

**Affiliations:** 1 Department of Nuclear Medicine, University Hospital of Munich, LMU Munich, Marchioninistr.15, 81377 Munich, Germany; 2 German Center for Neurodegenerative Diseases (DZNE), Munich, Germany; 3 Department of Neurology, University Hospital of Munich, LMU Munich, Munich, Germany; 4 Department of Neurosurgery, University Hospital, LMU Munich, Munich, Germany; 5 Department of Radiology, University Hospital of Munich, LMU Munich, Munich, Germany; 6 Department of Psychiatry and Psychotherapy, University Hospital of Munich, LMU Munich, Munich, Germany; 7 Life Molecular Imaging GmbH, Berlin, Germany; 8 Munich Cluster for Systems Neurology (SyNergy), Munich, Germany; 9 German Cancer Consortium (DKTK), Partner Site Munich, German Cancer Research Center (DKFZ), Heidelberg, Germany; 10 Institute of Clinical Neuroimmunology, University Hospital, LMU Munich, Munich, Germany; 11 Biomedical Center, Faculty of Medicine, LMU Munich, Munich, Germany; 12 Ageing Epidemiology (AGE) Research Unit, School of Public Health, Imperial College, London, UK; 13 Sheffield Institute for Translational Neuroscience (SITraN), University of Sheffield, Sheffield, UK; 14 Center for Neuropathology and Prion Research, LMU Munich, Munich, Germany

**Keywords:** MAO-B, PET, Astrocytes, Deprenyl

## Abstract

**Objectives:**

Reactive gliosis is a common pathological hallmark of CNS pathology resulting from neurodegeneration and neuroinflammation. In this study we investigate the capability of a novel monoamine oxidase B (MAO-B) PET ligand to monitor reactive astrogliosis in a transgenic mouse model of Alzheimer`s disease (AD). Furthermore, we performed a pilot study in patients with a range of neurodegenerative and neuroinflammatory conditions.

**Methods:**

A cross-sectional cohort of 24 transgenic (PS2APP) and 25 wild-type mice (age range: 4.3–21.0 months) underwent 60 min dynamic [^18^F]fluorodeprenyl-D2 ([^18^F]F-DED), static 18 kDa translocator protein (TSPO, [^18^F]GE-180) and β-amyloid ([^18^F]florbetaben) PET imaging. Quantification was performed via image derived input function (IDIF, cardiac input), simplified non-invasive reference tissue modelling (SRTM2, DVR) and late-phase standardized uptake value ratios (SUVr). Immunohistochemical (IHC) analyses of glial fibrillary acidic protein (GFAP) and MAO-B were performed to validate PET imaging by gold standard assessments. Patients belonging to the Alzheimer’s disease continuum (AD, *n* = 2), Parkinson’s disease (PD, *n* = 2), multiple system atrophy (MSA, *n* = 2), autoimmune encephalitis (*n* = 1), oligodendroglioma (*n* = 1) and one healthy control underwent 60 min dynamic [^18^F]F-DED PET and the data were analyzed using equivalent quantification strategies.

**Results:**

We selected the cerebellum as a pseudo-reference region based on the immunohistochemical comparison of age-matched PS2APP and WT mice. Subsequent PET imaging revealed that PS2APP mice showed elevated hippocampal and thalamic [^18^F]F-DED DVR when compared to age-matched WT mice at 5 months (thalamus: + 4.3%; *p* = 0.048), 13 months (hippocampus: + 7.6%, *p* = 0.022) and 19 months (hippocampus: + 12.3%, *p* < 0.0001; thalamus: + 15.2%, *p* < 0.0001). Specific [^18^F]F-DED DVR increases of PS2APP mice occurred earlier when compared to signal alterations in TSPO and β-amyloid PET and [^18^F]F-DED DVR correlated with quantitative immunohistochemistry (hippocampus: *R* = 0.720, *p* < 0.001; thalamus: *R* = 0.727, *p* = 0.002). Preliminary experience in patients showed [^18^F]F-DED V_T_ and SUVr patterns, matching the expected topology of reactive astrogliosis in neurodegenerative (MSA) and neuroinflammatory conditions, whereas the patient with oligodendroglioma and the healthy control indicated [^18^F]F-DED binding following the known physiological MAO-B expression in brain.

**Conclusions:**

[^18^F]F-DED PET imaging is a promising approach to assess reactive astrogliosis in AD mouse models and patients with neurological diseases.

**Supplementary Information:**

The online version contains supplementary material available at 10.1186/s12974-023-02749-2.

## Introduction

In recent years, a growing body of research has been dedicated to deciphering the involvement of non-neuronal cells in neurodegenerative diseases [1–3]. In this regard, Alzheimer’s disease (AD), as the most common cause of dementia is characterized by the “A-T-N” co-occurrence of beta-amyloid, hyperphosphorylated tau-fibrils, and neuronal loss [4, 5], but compelling evidence further suggests that neuroinflammation is another major hallmark of the disease. Dysregulated microglia and astroglia significantly contribute to the progression of AD and various other neurodegenerative diseases as well, often even predating the onset of cognitive decline and increase of other biomarkers [6–8]. Even though the temporal and causal relationship between these two glial entities and their various neuroprotective and neurotoxic subtypes remain to be further elucidated, in vivo detection of these cell types holds the potential to diagnose and intervene in the earliest disease stages. Astrocytes in particular have been proposed as an additional, inflammatory biomarker within the A–T–N scheme (I + or I-) to further improve the phenotyping and staging of the disease [9]. In response to different stimuli, this cell type acquires a reactive state by changing its function, morphology and gene expression profile [10]. As a result, many proteins are upregulated, some of which have been introduced as semi-specific biomarkers for astrogliosis. For example, increases of glial fibrillary acidic protein (GFAP) in various biofluids—albeit heterogeneously expressed by astrocytes—have been associated with both traumatic and neurodegenerative brain diseases as well as in healthy aging brains due to astrocyte hypertrophy and proliferation [11, 12]. Consequently, GFAP is currently used as a frequent marker of reactive astrocytes in brain tissue, blood and cerebrospinal fluid [10]. However, to date, no small molecular compounds have been established that selectively bind to GFAP when using more precise in-vivo imaging techniques, such as positron emission tomography (PET) [13]. It is for this reason that multiple radiotracers have so far targeted another semi-specific surrogate for reactive astrocytes, namely, monoamine oxidase B (MAO-B) [13]. This enzyme is predominantly located in the outer mitochondrial membrane of astrocytes and to a lesser extent in other cell types, such as serotonergic neurons, oligodendrocytes and microglia [13, 14]. Initial PET studies with the [^11^C] labeled MAO-B inhibitor Deprenyl have shown significantly higher binding in prodromal AD patients when compared to healthy controls [15]. However, the short half-life of [^11^C] (20.4 min) limits the use of these tracers to centers with on-site cyclotrons [16]. Therefore, [^18^F]fluorodeprenyl has recently been developed as a more practical alternative, showing comparable binding patterns to [^11^C]-based tracers while also reducing the trapping rate and increasing elimination from regions with low MAO-B expression due to deuteration (i.e., [^11^C]Deprenyl-D2). As such, [^18^F]fluorodeprenyl-D2 ([^18^F]F-DED) is dependent on MAO-B concentrations rather than a combination of MAO-B and blood flow [16]. Given promising first-in-human results that were recently described for imaging of reactive astrogliosis in AD [17, 18], we sought to target astrogliosis in-vivo in a transgenic mouse model of AD at various ages by means of [^18^F]F-DED and to provide histological validation using GFAP immunostaining. We further aimed to evaluate the clinical feasibility of [^18^F]F-DED in a pilot cohort of patients with various neurological diseases. To determine the temporal and spatial relationship with other established biomarkers of AD, we also aimed to directly compare the time course of astrogliosis in transgenic and wild-type mice with microgliosis and beta-amyloid burden using [^18^F]GE-180 and [^18^F]florbetaben, respectively.

## Materials and methods

### Radiochemistry

[^18^F]F-DED was synthesized on a Trasis AllinOne (Ans, Belgium) automated synthesis unit (ASU) consisting of 3 series-connected manifolds with a total of 18 valves. The manifolds were clamped into the correct position at the module. All reagents and materials were assembled on the pre-defined positions of the manifold (position 2: eluent, position 5: SAX cartridge, position 8: precursor solution, position 11: water bag, position 12: PBS bag, position 13: SPE cartridge, position 15: EtOH, position 16: HPLC solvent). The software prompts were followed, and manual intervention was necessary only during HPLC purification. No carrier added [^18^F]fluoride was produced via ^18^O(p, n)^18^F reaction by proton irradiation of ^18^O-enriched water and delivered to the activity inlet reservoir. The activity was then trapped on a Waters QMA Plus Light Carb cartridge and eluted into the reactor using the eluent solution (5 mg K_222_, 7.24 µL 1 M K_2_CO_3_, 300 µL H_2_O and 300 µL MeCN). After azeotropic drying, the precursor solution (2 mg Cl-Deprenyl-D2 in 600 µL MeCN) was transferred to the reactor, and the reaction mixture was heated at 120 °C for 20 min. The reaction mixture was quenched with HPLC solvent (4 mL) and purified via semi-preparative HPLC (Inertsil ODS-4 C18, 250 × 10 mm, 5 µm, 100 Å; isocratic elution with 60% (v/v) 5 mM NaOAc (pH 4)/40% (v/v) EtOH; flow: 5 ml/min; UV detection: 254 nm). The product peak was collected, diluted with water (48 mL) and passed through a Waters Oasis Plus Light HLB cartridge pre-conditioned with 10 ml EtOH and 10 ml water. The cartridge was rinsed with water (10 mL) and the radiolabeled product was eluted with 1 mL EtOH and diluted with phosphate buffered saline (11 mL). The formulated product solution was transferred to a dispenser and filtered through a Merck Cathivex-GV sterile filter. The product was obtained in a RCY of 15 ± 3.0% n.d.c. (*n* = 16) and a RCP of 98 ± 1.2% (*n* = 16). Specific activity was 267 ± 120 GBq/µmol (*n* = 7).

### Preclinical study overview

All preclinical experiments were performed in compliance with the National Guidelines for Animal Protection, Germany, with approval of the local animal care committee of the Government of Upper Bavaria and overseen by a veterinarian. The preclinical study was conducted in a cross-sectional design. Small-animal PET scans were performed using the three tracers [^18^F]F-DED (MAO-B), [^18^F]GE-180 (TSPO) and [^18^F]florbetaben (β-amyloid) in male wild-type and transgenic mice (PS2APP) using young (5–6 months), intermediate (11–13 months), and aged (18–20 months) animals (Table 1). Every animal underwent dynamic [^18^F]F-DED (0–60 min p.i.) PET scans and static PET scans using [^18^F]GE-180 (60–90 min p.i.) and [^18^F]florbetaben (30–60 min p.i.). Wild-type controls for [^18^F]GE-180 and [^18^F]florbetaben were obtained from the large standardized in-house database [19]. After the final PET session, mice were perfused using phosphate-buffered saline while deeply anaesthetized. The brain was fixed overnight with paraformaldehyde 4% before immunohistochemical analysis.

**Table 1:** Overview on animals and key results of the cross-sectional preclinical study

Mouse model	Age (mo)	[^18^F]F-DED small animal PET (*n*)	[^18^F]F-DED small animal PET (DVR_HIP_)	[^18^F]F-DED small animal PET (DVR_THA_)	[^18^F]GE-180 small animal PET (*n*)	[^18^F]florbetaben small animal PET (*n*)	
PS2APP	5.9	7	1.09 ± 0.07	1.17 ± 0.06*	7	6	
	13.4	9	1.11 ± 0.05*	1.18 ± 0.07	6	8	
	19.5	8	1.20 ± 0.05**	1.25 ± 0.07**	5	8	
Wild type	5.1	11	1.07 ± 0.03	1.09 ± 0.06			
	11.2	6	1.03 ± 0.07	1.08 ± 0.12			
	18.6	8	1.06 ± 0.06	1.08 ± 0.03			

Small-animal PET DVRs are shown for [^18^F]F-DED

Significant differences in PS2APP mice vs. age-matched wild-type controls are indicated by *p < 0.5; **p < 0.001

### Animals

Animals were housed in a temperature- and humidity-controlled environment with a 12-h light–dark cycle, with free access to food (Ssniff, Soest, Germany) and water. We used 25 male wild-type C57Bl/6 (WT) and 24 male PS2APP transgenic mice (TG) in this investigation. The transgenic B6.PS2APP (line B6.152H) is homozygous for both the human presenilin (PS) 2, N141I mutation and the human amyloid precursor protein (APP) K670N, M671L mutation. The APP and PS2 transgenes are driven by mouse Thy-1 and mouse prion promoters, respectively. This line had been created by co-injection of both transgenes into C57Bl/6 zygotes [20]. Homozygous B6.PS2APP (PS2APP) mice show first appearance of plaques in the cortex and hippocampus at 5–6 months of age [21].

### Small-animal PET data acquisition, reconstruction and coregistration

All PET procedures followed an established standardized protocol for acquisition, reconstruction, and post-processing [22–24]. In brief, [^18^F]F-DED (13.0 ± 2.5 MBq) with an emission window of 0–60 min p.i. was used to analyze MAO-B expression, [^18^F]GE-180 TSPO–PET (14.2 ± 2.6 MBq) with an emission window of 60–90 min p.i. was used to measure cerebral TSPO expression, and [^18^F]florbetaben β-amyloid-PET (11.1 ± 1.7 MBq) with an emission window of 30–60 min p.i. was used for assessment of fibrillary cerebral amyloidosis. Existing in-house normal cohorts of wild-type mice at matching ages were used for TSPO- and β-amyloid-PET [25, 26]. All analyses were performed by PMOD (V3.5, PMOD technologies, Basel, Switzerland). Spatial coregistration was performed with tracer specific templates as previously described [27].

### Small-animal PET data analyses

Hippocampus (24 mm^3^) and thalamus (27 mm^3^) were defined as target regions of interests and the cerebellum (56 mm^3^) was defined as a potential reference region based on the Mirrione atlas [28].

[^18^F]F-DED volume-of-distribution (V_T_) images were calculated with an image derived input function (IDIF) [29] using the methodology described by Logan et al. implemented in PMOD [30]. The plasma curve was obtained from a standardized VOI (3 mm sphere) placed in the left heart (including myocardium and ventricle). A maximum error of 10% and a V_T_ threshold of 0 were selected for modelling of the full dynamic imaging data. Cerebellar V_T_ were compared between young and aged mice from both genotypes to evaluate the suitability of the cerebellum as proposed pseudo-reference tissue. Distribution volume ratios (DVRs) of [^18^F]F-DED PET were subsequently calculated by simplified reference tissue 2 (SRTM2) modelling in PMOD, using the cerebellum as a reference tissue. DVRs served as the primary endpoint of [^18^F]F-DED PET analyses. SUV ratios (SUVR) of the 30–60 min p.i. time window were calculated with the cerebellum as a reference tissue to explore the suitability of simplified late-static imaging. TSPO- and β-amyloid PET were analogously quantified as SUVR with the same cerebellar reference tissue [23, 31].

### Immunohistochemistry

A total of 19 PS2APP mice and 10 wild-type mice, each divided into three age-related groups, were used for immunohistochemistry (wild-type mice: 5 months *n* = 3, 13 months *n* = 4, 19 months *n* = 3; PS2APP mice: 5 months *n* = 6, 13 months *n* = 10, 19 months *n* = 3). 50 µm thick slices were cut in a sagittal plane using a vibratome (VT1200S, Leica Biosystems). Four slices per animal, between the lateral coordinates 2.16 mm and 0.84 mm, were collected, containing the regions of interest: hippocampus, thalamus and cerebellum. Slices were treated with blocking solution (3% normal goat serum and 2% BSA in 0.3%Triton and PBS to a total volume of at least 200 µl per well/slice) for 3 h at RT. The following primary antibodies were used: rabbit anti-GFAP 1:500 (180063, Invitrogen by Thermo Fisher Scientific, California, USA), chicken anti-GFAP 1:500 (ab5541, Merck Millipore, Darmstadt, Germany), mouse anti-MAO-B 1:800 (D-6, sc-515354, Santa Cruz Biotechnology, Texas, USA), guinea pig anti-Iba1 1:500 (234004, Synaptic Systems, Göttingen, Germany), mouse anti-β-amyloid 1:500 (NAB228, sc-32277, Santa Cruz Biotechnology, Texas, USA) and goat anti-TPH2 1:500 (ab121013, Abcam, Cambridge, UK), diluted in blocking solution (1% normal goat serum and 1% BSA in 0.3% Triton and PBS to a total volume of at least 200 µl per well/slice), applied to the slices and incubated over-night at 4 °C on a horizontal shaker. Slices were washed three times, each 10 min with PBS to remove the spillover of the primary antibodies. Secondary antibodies, goat anti-rabbit Alexa Flour 488 (1:500), goat anti-chicken Alexa Fluor 488 (1:500), goat anti-guinea pig Alexa Fluor 555 (1:500), goat anti-mouse Alexa Fluor 647 (1:500) and donkey anti-goat Alexa Fluor 568 (1:500) diluted in blocking solution, were applied. Slices were incubated for 2 h at RT on a horizontal shaker, protected from light. Slices were washed 3 × 10 min with PBS and subsequently incubated in Sudan black for 15 min on a horizontal shaker at RT to reduce autofluorescence. After 3 × 10 min washing with PBS, slices were mounted and cover slipped with fluorescence mounting medium (Dako, Santa Clara, USA). Three-dimensional images were acquired with an Apotome microscope (Zeiss Oberkochen, Germany) using a 10×, 20× and 60× objective. The analysis programs Zeiss blue and ImageJ were used for quantification.

To analyze the MAO-B signal in GFAP positive astrocytes, two slices per animal were selected. From each of these slices, we acquired z-stack images (15 µm) in the hippocampus (CA1/Subiculum) and thalamus with a 60× objective. Each 4 cells per area got quantified as the perceptual area of MAO-B in GFAP-positive astrocytes. To this end, we created a mask of the GFAP-positive astrocyte and transferred it onto the MAO-B image. After a local brightness/contrast adjustment, we set a fixed threshold and used the “analyze particle” function to calculate the MAO-B area (%) inside the mask of the GFAP-positive astrocyte.

Plaque load (Aβ), astrogliosis (GFAP) and microgliosis (Iba1) were analyzed in the hippocampus/subiculum of 3 slices per animal by quantifying the total area (%) of each signal of an orthogonal projection of a 10 µm z-stack.

### Human PET imaging

Eight patients belonging to the AD continuum (*n* = 2), Parkinson’s disease (*n* = 2), multiple system atrophy (MSA, *n* = 2), autoimmune encephalitis (*n* = 1), oligodendroglioma (*n* = 1), and one healthy control received [^18^F]F-DED PET in a clinical setting at a tertiary center after giving written informed consent at the Dept. of Nuclear Medicine, LMU University Hospital. Patient characteristics are provided in Table 2. Toxicology was assessed in rats prior to the human study, indicating a No Observed Adverse Effect Level (NOAEL) of 33.33 µg/kg. The maximum injected mass was determined at 3.22 µg [^18^F]F-DED for a 60 kg patient and a 100-fold safety margin. The A–T–N status of patients belonging to the AD continuum was determined using PET (A: [^18^F]flutemetamol late-phase 90–110 min p.i.; T: [^18^F]PI-2620 30–60 min p.i.; N: either [^18^F]flutemetamol early phase 0–10 min p.i. or [^18^F]FDG 30–50 min p.i.) or CSF (A: Aβ_42/40_ ratio; T: p-Tau).

**Table 2:** Overview of the demographics of human subjects included in this study

Diagnosis	Age (years)	Gender	Disease duration (years)	[^18^F]F-DED dose (MBq)	Height (cm)	Body weight (kg)	Disease severity indices
MCI Alzheimer’s pathologic change (A + T − N −)	69	M	0.5	190	170	69	MMSE: 26/30
AD-Dementia (A + T + N +)	66	F	2.0	169	163	52	MMSE: 21/30
PD (1.7 y disease duration)	55	F	1.7	180	165	62	UPDRS III: 14; MoCA: 28/30
PD (4.8 y disease duration)	56	F	4.8	149	167	64	UPDRS III: 18; H&Y: 1; MoCA: 29/30
Autoimmune encephalitis	53	M	25.2	217	182	72	–
MSA-C	56	M	2.1	162	190	84	UPDRS III: 30; H&Y: 2; MoCA: 28/30
MSA-P	65	M	4.2	134	173	96	UPDRS III: 19; MoCA: 29/30
ODG	60	F	1.3	244	160	61	–
Healthy control	28	F	–	150	170	64	–

*MCI* mild cognitive impairment; *AD* Alzheimer’s disease, *A* β-amyloid; *T* tau; *N* neurodegeneration; *PD* Parkinson’s disease; *MSA* Multiple system atrophy; *C*  cerebellar subtype; *P* parkinsonian subtype; *ODG* Oligodendroglioma; *MMSE* mini mental state examination; *MoCA*  Montreal Cognitive Assessment; *UPDRS* Unified Parkinson’s Disease Rating Scale; *H&Y* Hoehn and Yahr scale

Caffeine consumption was prohibited starting 3 h before tracer injection. No patients with active smoking history were examined. After ldCT for attenuation correction, 177 ± 35 MBq [^18^F]F-DED were injected i.v. as a slow bolus injection (~ 10 s) followed by dynamic 60-min emission recording on harmonized Siemens PET/CT systems (Biograph 64, Biograph mCT). OSEM3D reconstruction was performed as previously described [32]. Data analysis was approved by the local ethics committee of the medical faculty of the LMU Munich (Application Number: 21-0721). Calculation of [^18^F]F-DED PET volume of distribution (V_T_) was performed using the average carotid tracer uptake as image-derived input function (IDIF) for non-invasive kinetic modeling. For proper quantitative representation of the tracer uptake in the brain, we calculated and compared four different kinetic models, since reversible and irreversible components of [^18^F]F-DED were not investigated in the human brain yet: a one-tissue compartment model (Logan plot), a one-tissue compartment model with two rate constants (1TC2k) as well as two-tissue compartment models with either three (2TC3k) or four rate constants (2TC4k) depending on the reversibility of the binding behaviour. We then calculated *F* tests to statistically compare the simplest model to the more complex models in each brain region. Selected target regions of the Hammers atlas consisted of the brainstem, the cerebellar white matter, a cortical composite, the mesial temporal lobe and the putamen. Spatial normalization and brain region definition was performed using PMOD’s PNEURO tool with existing MRI T1 MPRAGE sequences. Analysis of PET imaging data was performed visually and by volume-of-interest-(VOI)-based analysis of V_T_. In addition, static 30–60 min SUVr images and regional SUVr were evaluated as simplified quantification using a parietal lobe white matter reference tissue (predefined by the Hammers atlas) to assess relative signal changes in the investigated subjects. Time-activity SUV curves were inspected qualitatively.

### Statistics

Group comparisons of VOI-based small-animal PET results or immunohistochemical quantification were assessed by one-way ANOVA with Tukey post-hoc correction using Graph Pad Prism (V8). Two-way ANOVA was used when two categorical variables were included in the analysis. For correlation analyses, Pearson’s coefficient of correlation was calculated after controlling for normal distribution. Immunohistochemistry data were expressed as mean ± standard deviation. A threshold of *P* < 0.05 was considered to be significant for rejection of the null hypothesis.

## Results

### Suitability of the cerebellum as a pseudo-reference region for [^18^F]F-DED PET quantification in PS2APP and wild-type mice

**Fig. 1:**
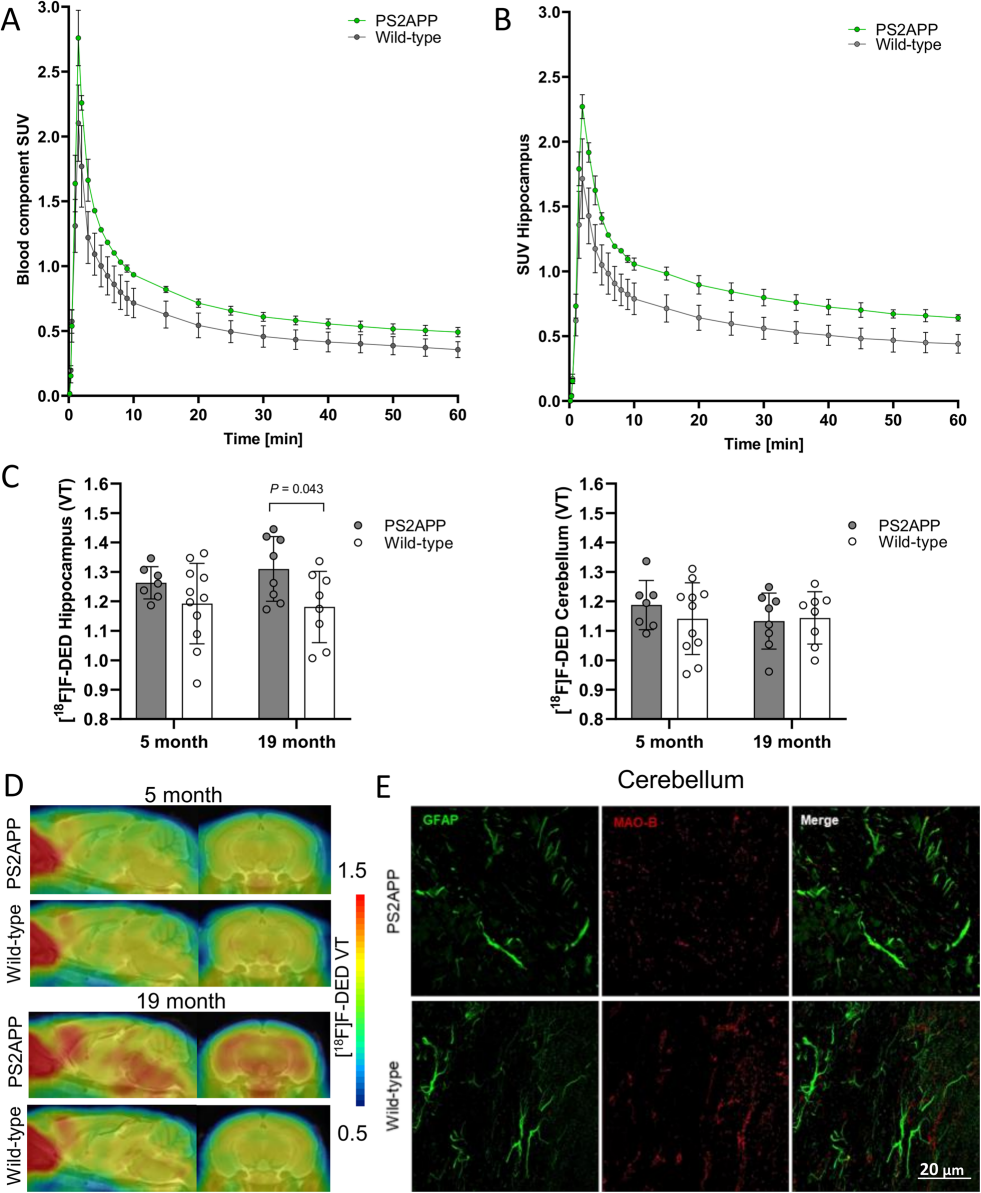
**A**, **B** [^18^F]F-DED cardiac blood input curves and hippocampal time-activity-curves in comparison of PS2APP (green) and wild-type (grey) mice. **C** Image-derived input function derived [^18^F]F-DED V_T_ in hippocampus and cerebellum of PS2APP and age matched wild-type mice at 5 and 19 months of age. **D** Coronal and sagittal slices of [^18^F]F-DED V_T_ in PS2APP and age-matched wild-type mice are illustrated upon a MRI template. **E** Immunohistochemistry of similar GFAP and MAO-B levels in the cerebellum of PS2APP and wild-type mice at 19 months of age

The [^18^F]F-DED blood input curve derived from the heart showed the expected sharp peak after 90 s and only low background activity in the late acquisition phase (Fig. 1A). Brain uptake of [^18^F]F-DED indicated a rapid influx and sufficient washout from brain parenchyma (Fig. 1B). IDIF derived [^18^F]F-DED V_T_ in hippocampus of PS2APP and wild-type mice were similar at 5 months of age (1.26 ± 0.06 vs. 1.19 ± 0.14; *p* = 0.216), but PS2APP mice had higher V_T_ at 19 months of age when compared to age-matched wild-type (1.31 ± 0.11 vs. 1.18 ± 0.12; *p* = 0.043; Fig. 1C, D). We observed no relevant difference in [^18^F]F-DED V_T_ of the cerebellum regardless of age and genotype (Fig. 1C, D). Immunohistochemistry of the cerebellum revealed only few GFAP positive cells with low MAO-B expression in PS2APP and wild-type mice (Fig. 1E).

### [^18^F]F-DED PET detects reactive astrogliosis in PS2APP mice

**Fig. 2:**
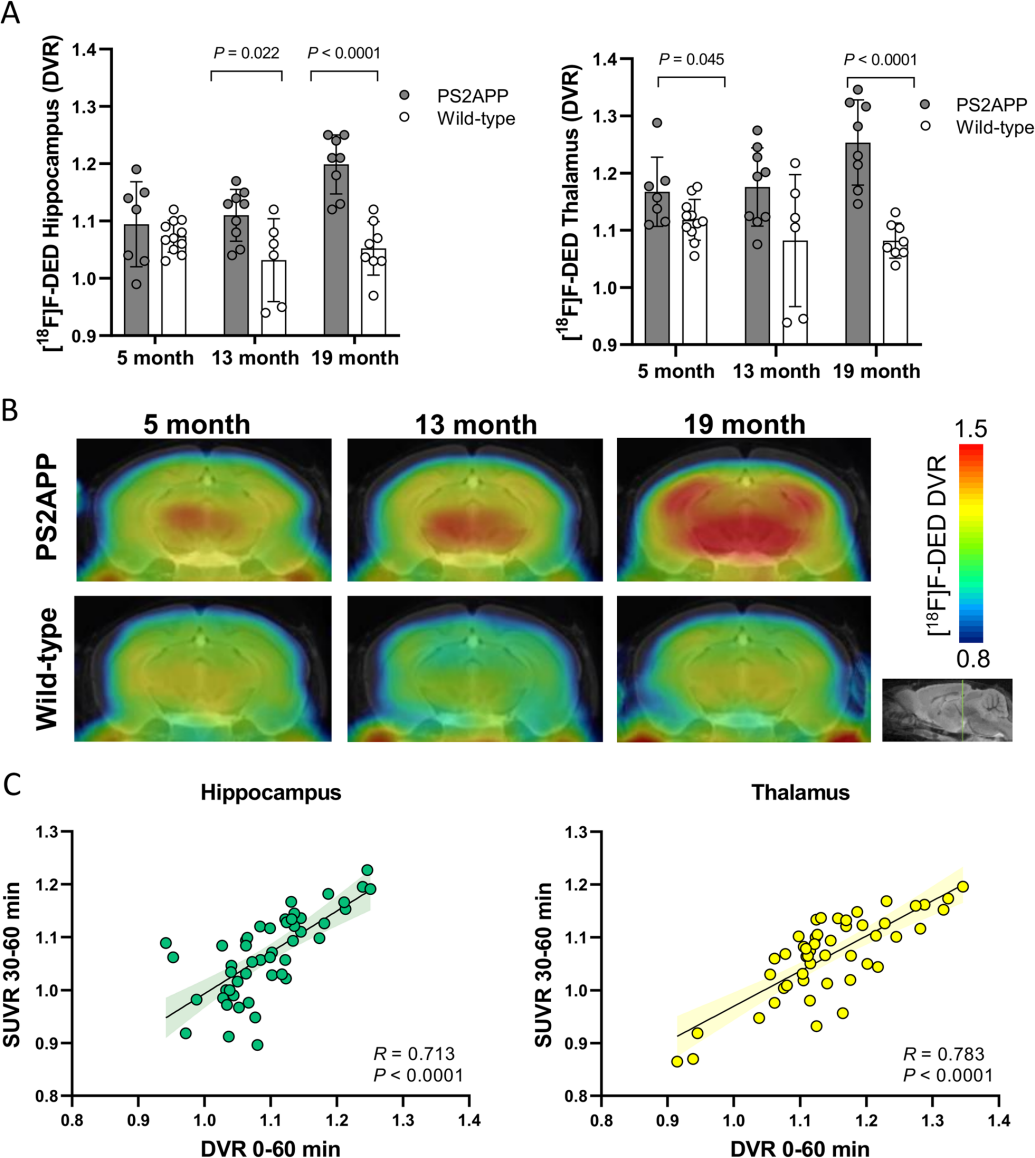
**A** Quantitative [^18^F]F-DED PET in comparison of PS2APP and age-matched wild-type mice animals at different ages. **B** Coronal planes of [^18^F]F-DED mean DVR maps at different ages of PS2APP animals and wild-type animals projected upon a MRI mouse atlas (gray scale). **C** Correlation of [^18^F]F-DED DVRs calculated from 60-min dynamic small-animal PET recordings with corresponding 30–60 min SUVR (reference region cerebellum). 95% confidence intervals are represented by color shaded areas

PS2APP mice suggested an age-depended increase of [^18^F]F-DED DVR in hippocampus (*p* = 0.003) and thalamus (*p* = 0.039), whereas wild-type mice did not show significant changes of [^18^F]F-DED DVR with age in hippocampus (*p* = 0.218) and thalamus (*p* = 0.372). PS2APP mice showed elevated hippocampal [^18^F]F-DED DVR when compared to age-matched wild-type mice at 13 months (+ 7.3%; *p* = 0.022) and 19 months of age (+ 12.3%; *p* < 0.0001; Fig. 2A, B). Thalamic [^18^F]F-DED DVR of PS2APP mice were significantly higher at 5 months (+ 4.3%; *p* = 0.045) and 19 months of age (+ 15.2%; *p* < 0.0001; Fig. 2A, B) when compared to age-matched wild-type mice. There was a strong positive correlation between 0 and 60 min DVR, and the 30–60 min static frame SUVr for hippocampus (*R* = 0.713; *p* < 0.0001) and thalamus (*R* = 0.783; *p* < 0.0001; Fig. 2C). [^18^F]F-DED quantification in the neocortex showed similar results when compared to hippocampus and thalamus (Additional file 1: Fig. S1).

**Fig. 3:**
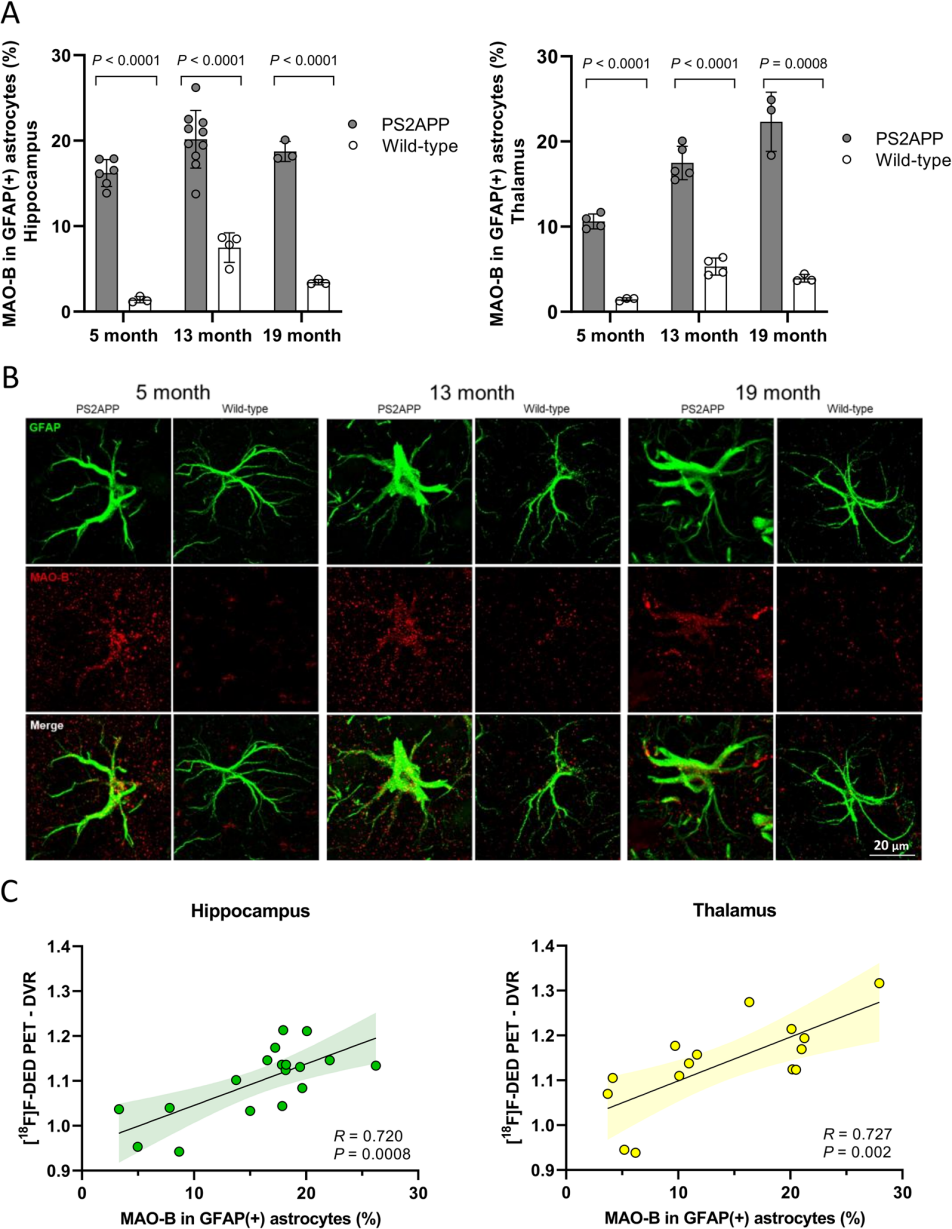
**A** MAO-B expression in GFAP-positive astrocytes (%) in comparison of PS2APP and age-matched wild-type mice together with **B** illustration of immunohistochemical staining in the hippocampus. **C** Correlation of [^18^F]F-DED PET DVRs with MAO-B in GFAP-positive astrocytes (%). 95% confidence intervals are represented by color shaded areas

Immunohistochemical staining for MAO-B expression in GFAP-positive astrocytes showed an early onset of elevated MAO-B expression in PS2APP mice when compared to wild-type mice which increased with age (Fig. 3A, B). Higher MAO-B expression with increase in GFAP-positive astrocytes of PS2APP mice of all age-related groups was observed when compared to their age-matched wild-type controls in hippocampus (all *p* < 0.001) and thalamus (all *p* < 0.01). MAO-B in GFAP-positive astrocytes showed a strong positive correlation with the [^18^F]F-DED uptake in vivo in hippocampus (*R* = 0.720; *p* < 0.001) and in thalamus (*R* = 0.727; *p* = 0.002; Fig. 3C).

### Hippocampal MAO-B PET signal as a potential early biomarker in PS2APP mice

**Fig. 4:**
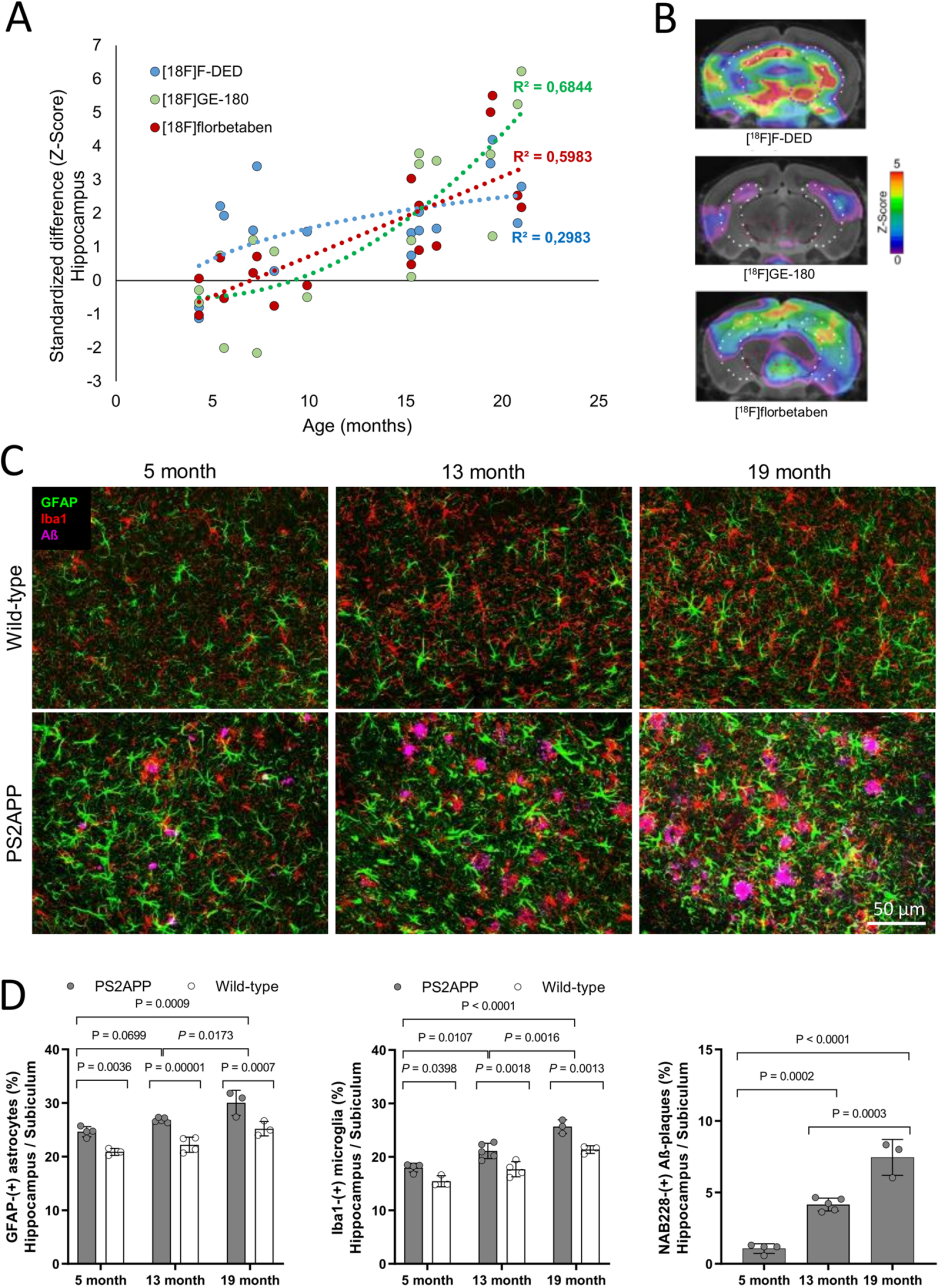
**A** Life-course kinetics for PS2APP mice as expressed by standardized differences (z-score) in hippocampus for the 3 radiotracers. **B** Coronal planes of z-scores for the three radiotracers. **C** Sagittal plane of the hippocampus/subiculum of wild type and PS2APP mice stained against GFAP, Iba1 and Aβ at 5, 13 and 19 months of age. **D** Mean (± SD) GFAP, Iba1 and Aβ staining (area-%) for PS2APP animals at different ages compared to age-matched wild-type animals

To compare trajectories of reactive astrocytes, activated microglia and β-amyloidosis, we calculated standardized differences of tracer signals in the hippocampus of PS2APP mice (vs. WT) for [^18^F]F-DED, [^18^F]GE-180 and [^18^F]florbetaben. [^18^F]F-DED as a function of age indicated a logarithmic fit (*y* = 1.33ln(*x*)−1.50; *R*^2^ = 0.298), whereas [^18^F]GE-180 (*y* = 0.02*x*^2^−0.15*x*−0.20; *R*^2^ = 0.684) and [^18^F]florbetaben (*y* = 0.01*x*^2^−0.05*x*−0.28; *R*^2^ = 0.621) indicated exponential fits (Fig. 4A, B). Standardized differences between 4.3 and 9.9 months of age were higher for [^18^F]F-DED when compared to [^18^F]GE-180 (p = 0.044) and [^18^F]florbetaben (*p* = 0.039). Immunohistochemistry confirmed astrogliosis in PS2APP mice starting as early as 5 months, accompanying the Aβ-plaque formation, which occurs first in the hippocampus/subiculum and spreads to the cortex and thalamus with increasing age (Fig. 4C, D; Additional file 1: Fig. S2). In PS2APP mice at 5 months of age, visual and quantitative increases of GFAP(+) astrocytes exceeded the increases of Iba1( +) microglia (Fig. 4C, D).

### Initial clinical experience with [^18^F]F-DED PET matches expected magnitude and topology of reactive astrogliosis in patients with neurological diseases

**Fig. 5:**
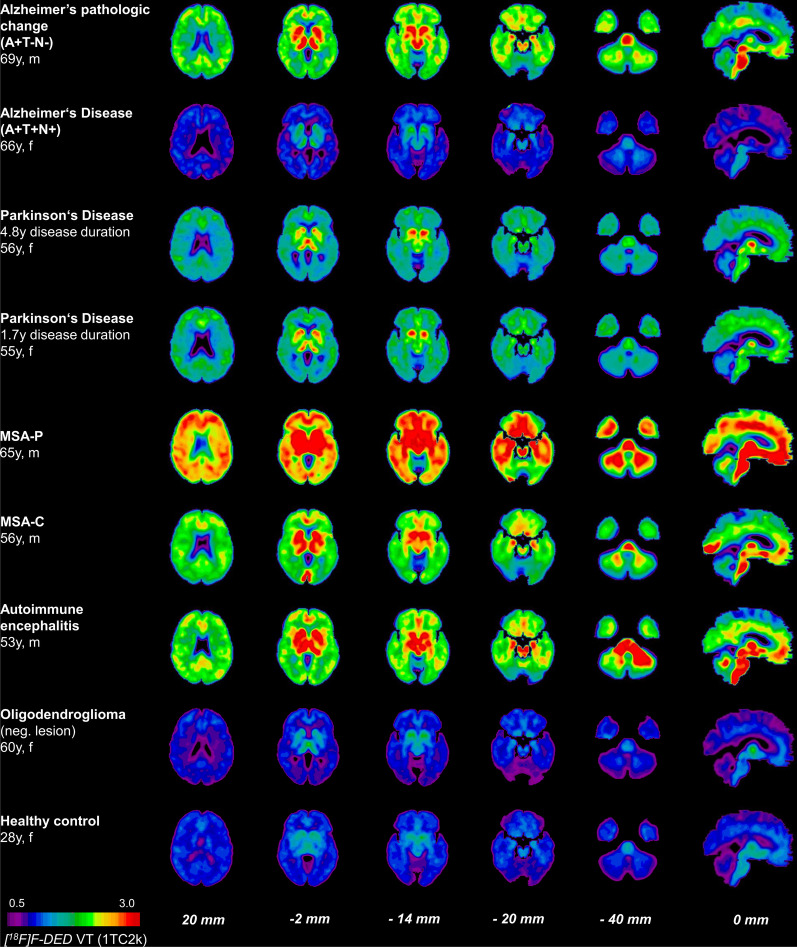
Axial and sagittal planes show [^18^F]F-DED volumes of distribution (V_T_) based on the 1TC2k compartment model at levels of neocortical regions, basal ganglia, hippocampus, cerebellum (all coronal) and brainstem (sagittal). The lesions of patients with autoimmune encephalitis and oligodendroglioma are indicated with white arrows. ATN indicates biomarker positivity for β-amyloid, tau and neurodegeneration in patients belonging to the AD continuum. MSA = multiple systems atrophy

Based on the promising preclinical data, [^18^F]F-DED imaging was translated to the clinical stage. [^18^F]F-DED was administered to eight patients and one healthy control (Table 2). The mean and standard deviation of the administered mass of [^18^F]F-DED was 0.074 ± 0.057 µg (range, 0.020–0.197 µg). The mean administered activity was 177 ± 35 MBq (range, 134–244 MBq). There were no adverse or clinically detectable pharmacologic effects in any of the nine subjects and no significant changes in vital signs. The injection and PET/CT procedures were well-tolerated in all participants. In the kinetic modeling analyses, a slightly superior fit of the 2TC3k model over the 1TC2k model was observed in the brainstem, the medio-temporal lobe and the putamen, however, driven by a single patient with exceptionally high fitting values. There was also no further improvement when comparing the 2TC4k model with the 2TC3k model, since a fourth parameter to the kinetic model did not lead to a significant reduction in the variation of residuals (Additional file 1: Table S1). We, therefore, concluded that the 1TC2k model represents human [^18^F]F-DED PET data best and generated V_T_ images based on that model (see Fig. 5). In addition, we validated the agreement between V_T_ values based on the 1TC2k compartment model and Logan Plot by computing Bland–Altman as well as scatter plots which showed no significant over- or underestimation for either method of quantification (Additional file 1: Fig. S3). A visual comparison of the quantitative V_T_ values regions confirmed this high level of concordance in all five target regions (Additional file 1: Table S2).

[^18^F]F-DED PET V_T_ and SUVr images corresponded to the expected patterns of MAO-B expression in brain and to the phenotype of the examined patients (Fig. 5, Additional file 1: Figs. S4–S6). The patient with Alzheimer’s pathologic change that did not yet show evidence of tau pathology ([^18^F]PI-2620 PET) and who had no significant neurodegeneration as assessed by β-amyloid-PET perfusion imaging (A + T-N-), revealed high cortical and subcortical [^18^F]F-DED binding with regional predominance in cortical AD signature regions (Fig. 5; Additional file 1: Figs. S4–S7). Relatively low [^18^F]F-DED binding was observed in the patient with AD and who had evidence for the presence of β-amyloid and tau pathology (both assessed in CSF) and neurodegeneration as assessed by [^18^F]FDG–PET (A + T + N + ; Fig. 5; Additional file 1: Fig. S7). In Parkinson’s disease, moderate global [^18^F]F-DED binding with pronounced signal in the basal ganglia was observed in two patients with 1.7 and 4.8 year disease duration. Both patients with MSA had much stronger [^18^F]F-DED binding in cortical and subcortical brain regions when compared to patients with PD (MSA-P > MSA-C > PD) with predominance in basal ganglia (MSA-P), pons (both) and cerebellar white matter (MSA-C) and with a higher cerebellum-to-putamen-ratio in the patient with MSA-C (0.81) when compared to MSA-P (0.73; Fig. 5; Additional file 1: Fig. S7). The patient with autoimmune encephalitis had a strong [^18^F]F-DED binding with predominance in the cerebellar peduncles and the left cerebellar deep white matter, matching but also extending the lesions on MRI (Fig. 5; Additional file 1: Fig. S7). The patient with oligodendroglioma was [^18^F]F-DED negative at the lesion site (right frontal cortex) and indicated regional [^18^F]F-DED binding that followed physiological MAO-B expression in healthy brain, such as the healthy control (Fig. 5) [17, 18]. [^18^F]F-DED V_T_ images based on Logan Plot analysis revealed matching regions of elevated [^18^F]F-DED binding (Additional file 1: Fig. S4).

Time-activity-curves showed fast brain uptake and a region dependent washout until 10–20 min p.i.. The cerebellar white matter indicated further moderate washout > 20 min p.i., whereas [^18^F]F-DED binding in all target regions indicated stable (irreversible) binding in the late acquisition phase (Additional file 1: Fig. S8).

## Discussion

In recent years, several PET tracers have been developed that target MAO-B, a surrogate for reactive astrocytes and, therefore, a promising new biomarker for neuroinflammation in various neurological diseases. However, most of these ligands have so far been based on [^11^C] [33–35], whose short half-life (20.4 min) limits its use in clinical settings to centers with on-site cyclotrons. Consequently, an alternative radiolabeling has recently been carried out on the basis of [^18^F], which possesses a much longer half-life of 109.8 min and is, therefore, more suitable for broad clinical use. Adding to that, the deuteration of [^18^F]F-DED overcomes previous issues concerning high background signaling by enabling a better wash-out from regions low in MAO-B expression [16], thus yielding excellent tracer properties for neuroimaging in both rodents and humans. Even though in the present study, we were not able to directly provide evidence for the binding specificity of [^18^F]F-DED, initial work done by Nag et al. (2015) showed high in vitro binding of this radiotracer to regions with increased levels of MAO-B in the monkey brain. In addition, blocking of MAO-B with L-deprenyl successfully suppressed any [^18^F]F-DED binding thereafter [16]. We, therefore, based our work on the assumption that this tracer targets MAO-B with adequate specificity. With regard to radiometabolites, we were not able to obtain a direct analysis of arterial blood in the current study. Here, too, we based our assumption on previous findings indicating that [^18^F]F-DED is partly metabolized and has a similar retention time when compared to the less lipophilic metabolites [16]. However, even though this byproduct could likely contaminate [^18^F]F-DED PET signal, only about 5% of overall radioactivity was previously attributed to [^18^F]flurometamphatamine-D2 [16]. Hence, we assumed that a sufficiently high proportion of our [^18^F]F-DED signal represents the parent compound and, therefore, aimed to investigate the potential use of this radiotracer as a biomarker for early neuroinflammation in both transgenic mice as well as a human pilot sample.

The first part of our study consisted of a cross-sectional investigation of [^18^F]F-DED PET in the PS2APP β-amyloid mouse model in conjunction with semi-quantitative immunohistochemical validation. Our preclinical findings showed an age-dependent increase of [^18^F]F-DED signal in the hippocampus and thalamus in PS2APP mice. These transgenic mice typically start expressing β-amyloid plaques at about 5–6 months of age [21], with a close correlation to TSPO expression during the later life course [23]. Similarly, reactive astrogliosis in PS2APP showed a close topological association with β-amyloid pathology during aging with highest abundance in hippocampus, thalamus and cortex. Increasing MAO-B PET signals with age in transgenic models of β-amyloid pathology are in line with a previous study using [^11^C]DED in APP_ArcSwe_ mice compared to wild-type mice [36]. However, another study using this tracer in APP_Swe_ mice found increased MAO-B PET signals before onset of fibrillary β-amyloid pathology compared to wild type but decreases of cortical and hippocampal [^11^C]DED signals towards wild-type levels during aging [7]. Thus, longitudinal trajectories of MAO-B PET signals may depend on the used mouse model and specific characteristics of their β-amyloid pathology which deserves head-to-head comparison with equal methodology in future studies. Interestingly, in our study, we found a [^18^F]F-DED signal elevation that preceded those of other AD biomarkers, showing earlier elevation and a plateau (logarithmic vs. exponential function) when compared to TSPO–PET and β-amyloid-PET signal. Keeping potential differences in both the sensitivity of the tracer and the target abundance in mind, this supports the assumption of an early astrocyte burst [37] in the pathophysiology of AD and substantiates the value of imaging astrogliosis as an early AD biomarker. In line, elevated GFAP levels were observed in plasma of early and late onset AD [38], but also in β-amyloid-positive cognitively normal individuals when compared to β-amyloid-negative controls [39]. Furthermore, [^18^F]F-DED signal strongly correlated with MAO-B expression in GFAP-positive astrocytes in our study, which strengthens the assumption that this radiotracer detects MAO-B positive reactive astrogliosis in a proportional manner. Importantly, the correlation of MAO-B PET signals with MAO-B positivity in GFAP( +) astrocytes allowed to correlate PET signal increases specifically with the claimed astrocytic source. We note that previous studies did not consistently report co-localized MAO-B and GFAP expression in immunohistochemistry [36], which indicates that our validated antibody could have superior sensitivity to astrocytic MAO-B expression.

Our preclinical study was also used to investigate the cerebellum as a potential reference tissue for simplified [^18^F]F-DED quantification, as previous studies have revealed that this region expresses only low amounts of MAO-B and shows only low ligand uptake [15, 40]. Adding to that, this tissue is also often used for standardization of other radiotracers used in this mouse model [23]. However, defining a proper reference region, i.e., one with no specific radiotracer uptake, is not entirely possible with MAO-B due to it being ubiquitously expressed throughout the brain (Additional file 1: Fig. S9). We, therefore, decided to normalize our preclinical [^18^F]F-DED data by defining a pseudo-reference region with comparatively low specific radiotracer uptake. This was indeed the case for the cerebellum, for which we did not find any significant differences in [^18^F]F-DED V_T_ signal between transgenic and wild-type mice as well as similarly low staining patterns of GFAP. Thus, we concluded that specific uptake was not affected by diseases status or age and that this region could, therefore, serve as a suitable pseudo-reference for our quantitative analyses (Fig. 1C, E).

Based on our previous experience with [^18^F]THK-5351 in MSA and PD [41], we further applied [^18^F]F-DED in a pilot sample of patients and one healthy control, with the rationale to detect globally and regionally increased MAO-B expression. Preliminary data of our first-in-human [^18^F]F-DED sample matched the expected binding magnitude and topology of MAO-B expression in various neurological diseases, although no generalized conclusions can yet be drawn due to the low number of cases. The topology in the healthy control and in regions with assumed low neuropathology matched the expression of MAO-B in healthy brain with highest abundance in the basal ganglia and low abundance in the cerebellum [17, 18]. Increased MAO-B expression in early stages of the AD continuum which were ameliorated at later stages were previously described for [^11^C]DED [15]. In line with the hypothesis of an astrocyte burst in early AD, we found strong [^18^F]F-DED binding with predominance in parietal and temporal AD signature regions when examining one β-amyloid-positive MCI patient that had no evidence of tau pathology and neurodegeneration yet. Contrary, [^18^F]F-DED binding was only low in the second patient with AD that had elevation of p-tau and widespread FDG hypometabolism. [^18^F]F-DED binding in two patients with PD indicated low and similar MAO-B expression at 1.7 and 4.8 year disease duration, whereas previous observations with the imidazoline 2 astrocyte tracer [^11^C]BU99008 showed a decline at later stages [42]. [^18^F]F-DED PET in both examined patients with MSA was higher when compared to PD and congruent with the phenotype, indicating pronounced binding in cerebellar white matter and pons in presence of clinical cerebellar predominance and pronounced binding in the putamen in MSA-P. A broader range of patients with typical and atypical Parkinsonian syndromes needs to be investigated with [^18^F]F-DED to allow conclusions on specific binding characteristics. Assessment of astrogliosis could also reflect a useful diagnostic biomarker in MSA, since MAO-B expression levels of patients with MSA exceeded the levels of patients with PD and healthy controls in autopsy [43]. Strong [^18^F]F-DED binding was also observed in a patient with autoimmune encephalitis with predominant cerebellar involvement, indicating astrocytosis/reactive astrogliosis. Thus, [^18^F]F-DED PET could be an interesting additional tool for assessment of the regional extent and the lesion activity in patients with inflammatory diseases of the CNS. The patient with oligodendroglioma had lower [^18^F]F-DED binding in the lesion when compared to cortical regions. Thus, the signal in brain tissue outside the lesion may serve as a preliminary pseudo control of normal MAO-B expression together with the young healthy control investigated. Taken together, these first-in-human [^18^F]F-DED data revealed MAO-B expression patterns of interest that may serve differential diagnosis of various neurodegenerative diseases. However, given the small sample size of the human cohort and lacking controls these observations should be interpreted with caution. Confirmation in a larger cohort and longitudinal follow-up imaging is warranted. The [^18^F]F-DED V_T_ obtained by an exploratory IDIF encourage further validation by arterial sampling including metabolite correction.

## Conclusion

In vivo imaging by PET with the novel MAO-B tracer [^18^F]F-DED indicates a potential for assessment of reactive astrogliosis in AD mouse models and patients with neurological diseases. [^18^F]F-DED binding correlates with immunohistochemical gold standard assessment and our results suggest that [^18^F]F-DED could be used as an early biomarker for neuroinflammation in this transgenic AD mouse model. Translation into preliminary human data showed regional congruence between tracer signal and expected disease topology, which indicates the potential for detection of regionally altered MAO-B expression.

## Supplementary Information

**Additional file 1:** **Table S1.** Kinetic modelling overview in the human cohort**.** A superior fit (*) of the 2TC3k compared to the 1TC2k model was found in three target regions due to high values in the autoimmune encephalitis (AIE) patient. *F* tests indicate that the more complex 2TC4k model does not lead to a further significant reduction in the variation of residuals. AIC = Akaike Information Criterion; SC = Schwartz Information Criterion; *χ²* = Sum of Squares of the weighted residuals divided by the degrees of freedom; AD = Alzheimer’s disease continuum; PD = Parkinson’s disease; MSA = multiple systems atrophy; ODG = oligodendroglioma. **Table S2.** Quantitative comparison of Volumes of distribution (V_T_) generated on the basis of a 1TC2k compartmental and a Logan Plot. Intraindividual comparison reveals similar values across all target regions for both quantification methods. AD = Alzheimer’s disease continuum; PD = Parkinson’s disease; MSA = multiple systems atrophy; AIE = autoimmune encephalitis; ODG = oligodendroglioma. **Figure S1.** (A) Mean (± SD) distribution volume ratios (DVRs) of [^18^F]F-DED PET for PS2APP animals at different ages compared to age-matched wild-type animals for the target region cortex. Significant differences between genotypes per timepoint are indicated. (B) Correlation of [^18^F]F-DED DVRs calculated from 60-min dynamic small-animal PET recordings with corresponding 30–60-min SUVR (reference region cerebellum). 95% confidence intervals are represented by dotted lines. **Figure S2.** Sagittal plane showing the cortex, hippocampus and thalamus stained against GFAP for astrocytes and Aβ (NAB228) for Aβ plaques in PS2APP mice at 5, 13 and 19 months of age. Plaques start to from in the subiculum at 5 months of age and spread to the cortex and thalamus. The plaque load is accompanied by astrogliosis which also starts 5 months and increases in an age-related manner. **Figure S3. **Bland–Altman plots (first row) comparing V_T_ values based on compartmental and Logan Plot analyses. The red line corresponds to the mean difference of V_T_ values, the black lines indicate the limits of agreement (Mean ± 1.96 * SD). Correlational analyses (second row) reveal high correlations between V_T_ values based on the 1TC2k and those based on Logan Plot. **Figure S4. **Axial and sagittal planes show [^18^F]F-DED Volumes of distribution (V_T_) based on a Logan Plot at levels of neocortical regions, basal ganglia, hippocampus, cerebellum (all coronal) and brainstem (sagittal). The lesions of patients with autoimmune encephalitis and oligodendroglioma are indicated with white arrows. **Figure S5. **Axial and sagittal planes show [^18^F]F-DED standardized uptake value ratios (SUVr) based on a parietal white matter reference region and a 30–60-min time frame at levels of neocortical regions, basal ganglia, hippocampus, cerebellum (all coronal) and brainstem (sagittal). Scaling is optimized to evaluate signal changes in the basal ganglia and brainstem regions. Parietal and temporal Alzheimer’s disease signature regions are indicated with pink arrows. Regions of interest in patients with Parkinson’s disease and multiple systems atrophy (MSA) are indicated with orange arrows. The lesions of the patient with autoimmune encephalitis are indicated with white arrows. **Figure S6. **Axial and sagittal planes show [^18^F]F-DED standardized uptake value ratios (SUVr) based on a parietal white matter reference region and a 30–60-min time frame at levels of neocortical regions, basal ganglia, hippocampus, cerebellum (all coronal) and brainstem (sagittal). Scaling is optimized to evaluate signal changes in the cortical regions. Parietal and temporal Alzheimer’s disease signature regions are indicated with pink arrows. Regions of interest in patients with Parkinson’s disease and multiple systems atrophy (MSA) are indicated with orange arrows. The lesions of the patient with autoimmune encephalitis are indicated with white arrows. **Figure S7. **Additional imaging characteristics of investigated patients of the Alzheimer’s disease (AD) continuum, multiple systems atrophy (MSA) and autoimmune encephalitis as assessed by β-amyloid-PET, tau-PET, FDG–PET and MRI. **Figure S8.** [^18^F]F-DED time-activity-curves (TACs) of patients with neurodegenerative diseases and multiple sclerosis in contrast to a patient with MAO-B negative oligodendroglioma. All TACs are scaled to the maximum SUV to allow direct comparison of the wash-out phase. MSA-P = Multiple systems atrophy–parkinsonian subtype; MSA-C = Multiple systems atrophy–cerebellar subtype; ODG = Oligodendroglioma; PD = Parkinson’s disease; AD = Alzheimer’s disease (A = amyloid-β; T = tau; N = neurodegeneration); SUV = standard-uptake-value; min = minutes. **Figure S9.** (**A**) MAO-B expression in GFAP-(+) astrocytes, (**B**) TPH2-(+) serotonergic neurons of the raphe nucleus and (**C**) in endothelia cells of blood vessels of wild type and PS2APP mice.

## Data Availability

All raw data can be obtained by the corresponding author upon reasonable request.
