# Supplementary material for: [18F]F-DED PET imaging of reactive astrogliosis in neurodegenerative diseases: preclinical proof of concept and first-in-human data
Source: J Neuroinflammation. 2023 Mar 11;20:68. doi: 10.1186/s12974-023-02749-2 (PMC10007845; doi:10.1186/s12974-023-02749-2)
Supplement: Supplementary file 1 — Additional file 1: Table S1. Kinetic modelling overview in the human cohort. A superior fit (*) of the 2TC3k compared to the 1TC2k model was found in three target regions due to high values in the autoimmune encephalitis (AIE) patient. F tests indicate that the more complex 2TC4k model does not lead to a further significant reduction in the variation of residuals. AIC = Akaike Information Criterion; SC = Schwartz Information Criterion; χ² = Sum of Squares of the weighted residuals divided by the degrees of freedom; AD = Alzheimer’s disease continuum; PD = Parkinson’s disease; MSA = multiple systems atrophy; ODG = oligodendroglioma. Table S2. Quantitative comparison of Volumes of distribution (VT) generated on the basis of a 1TC2k compartmental and a Logan Plot. Intraindividual comparison reveals similar values across all target regions for both quantification methods. AD = Alzheimer’s disease continuum; PD = Parkinson’s disease; MSA = multiple systems atrophy; AIE = autoimmune encephalitis; ODG = oligodendroglioma. Figure S1. (A) Mean (± SD) distribution volume ratios (DVRs) of [18F]F-DED PET for PS2APP animals at different ages compared to age-matched wild-type animals for the target region cortex. Significant differences between genotypes per timepoint are indicated. (B) Correlation of [18F]F-DED DVRs calculated from 60-min dynamic small-animal PET recordings with corresponding 30–60-min SUVR (reference region cerebellum). 95% confidence intervals are represented by dotted lines. Figure S2. Sagittal plane showing the cortex, hippocampus and thalamus stained against GFAP for astrocytes and Aβ (NAB228) for Aβ plaques in PS2APP mice at 5, 13 and 19 months of age. Plaques start to from in the subiculum at 5 months of age and spread to the cortex and thalamus. The plaque load is accompanied by astrogliosis which also starts 5 months and increases in an age-related manner. Figure S3. Bland–Altman plots (first row) comparing VT values based on compartmental and Logan Plo [file 12974_2023_2749_MOESM1_ESM.docx]

**Additional file 1 to “[^18^F]F-DED PET Imaging of Reactive Astrocytosis in Neurodegenerative Diseases: Preclinical Proof of Concept and First in Human Data”**

A. Ballweg*, C. Klaus*, L. Vogler*, S. Katzdobler, K. Wind, A. Zatcepin, S. Ziegler, B. Secgin, F. Eckenweber, B. Bohr, A. Bernhardt, U. Fietzek, B.-S. Rauchmann, S. Stoecklein, S. Quach, L. Beyer, M. Scheifele, M. Simmet, E. Joseph, S. Lindner, I. Berg, N. Koglin, A. Mueller, A.W. Stephens, P. Bartenstein, J.C. Tonn, N.L. Albert, T. Kümpfel, M. Kerschensteiner, R. Perneczky, J. Levin, L. Paeger, J. Herms, M. Brendel

**Tables**

|  |  | **1TC2k** | | | **2TC3k** | | | **2TC4k** | | | **F-Test** | | |
| --- | --- | --- | --- | --- | --- | --- | --- | --- | --- | --- | --- | --- | --- |
| *VOI* | Dx | *AIC* | *SC* | *χ²* | *AIC* | *SC* | *χ²* | *AIC* | *SC* | *χ²* | **1TC2k vs. 2TC3k** | **1TC2k vs. 2TC4k** | **2TC3k vs. 2TC4k** |
| Brainstem | MSA-C | 92.21 | 95.33 | 13.19 | 57.66 | 62.32 | 4.79 | 97.34 | 102.56 | 14.09 | *2.67* | *0.88* | *0,33* |
|  | MSA-P | 70.58 | 73.69 | 7.11 | 48.25 | 52.91 | 3.66 | 51.26 | 56.48 | 3.78 | *1.88* | *1.77* | *0,94* |
|  | AD | 70.40 | 73.51 | 7.07 | 72.47 | 77.13 | 7.31 | 75.39 | 80.61 | 7.52 | *0.94* | *0.88* | *0,94* |
|  | AD | 65.22 | 68.33 | 6.10 | 55.44 | 60.10 | 4.49 | 58.45 | 63.68 | 4.64 | *1.32* | *1.24* | *0,94* |
|  | AIE | 143.71 | 146.82 | 57.43 | -0.45 | 4.21 | 0.91 | 2.63 | 7.86 | 0.94 | *61.22* | *57.31* | *0,94* |
|  | PD | 59.13 | 62.24 | 5.12 | 60.61 | 65.27 | 5.21 | 64.11 | 69.34 | 5.45 | *0.95* | *0.88* | *0,92* |
|  | ODG | 72.07 | 75.18 | 7.42 | 74.06 | 78.73 | 7.65 | 77.07 | 82.30 | 7.90 | *0.94* | *0.88* | *0,94* |
|  | PD | 73.84 | 76.95 | 7.80 | 56.50 | 61.17 | 4.63 | 78.88 | 84.10 | 8.31 | *1.63* | *0.88* | *0,54* |
| **mean** | | **80,89** | **84.01** | **13.90** | **53.07** | **57.73** | **4.83** | **63.14** | **68.36** | **6.58** | **2.79*** | **1.99*** | **0.71** |
| CBW | MSA-C | 84.74 | 87.85 | 10.65 | 65.48 | 70.14 | 5.98 | 89.86 | 95.08 | 11.38 | *1.73* | *0.88* | *0,51* |
|  | MSA-P | 65.84 | 68.95 | 6.21 | 50.90 | 55.56 | 3.94 | 71.17 | 76.39 | 6.67 | *1.53* | *0.87* | *0,57* |
|  | AD | 90.31 | 93.42 | 12.49 | 92.25 | 96.92 | 12.86 | 95.15 | 100.37 | 13.23 | *0.94* | *0.89* | *0,94* |
|  | AD | 64.53 | 67.64 | 5.98 | 66.44 | 71.10 | 6.15 | 70.33 | 75.55 | 6.51 | *0.94* | *0.86* | *0,91* |
|  | AIE | 130.91 | 134.03 | 39.84 | 21.59 | 26.25 | 1.71 | 24.66 | 29.88 | 1.77 | *22.63* | *21.20* | *0,94* |
|  | PD | 84.91 | 88.02 | 10.70 | 86.92 | 91.58 | 11.04 | 90.67 | 95.90 | 11.65 | *0.94* | *0.86* | *0,92* |
|  | ODG | 86.11 | 89.22 | 11.08 | 88.20 | 92.87 | 11.45 | 91.09 | 96.31 | 11.78 | *0.94* | *0.88* | *0,94* |
|  | PD | 71.09 | 74.20 | 7.21 | 69.10 | 73.77 | 6.64 | 76.14 | 81.36 | 7.69 | *1.05* | *0.88* | *0,84* |
| **mean** | | **84,81** | **87.92** | **13.02** | **67.61** | **72.28** | **7.47** | **76.13** | **81.36** | **8.83** | **1.69** | **1.38** | **0.82** |
| Comp. Cortical | MSA-C | 87.67 | 90.79 | 11.58 | 86.45 | 91.12 | 10.89 | 93.07 | 98.29 | 12.47 | *1.03* | *0.87* | *0,85* |
|  | MSA-P | 64.33 | 67.44 | 5.94 | 65.10 | 69.76 | 5.92 | 69.77 | 74.99 | 6.41 | *0.97* | *0.87* | *0,89* |
|  | AD | 76.21 | 79.32 | 8.35 | 78.24 | 82.91 | 8.62 | 81.19 | 86.42 | 8.88 | *0.94* | *0.88* | *0,94* |
|  | AD | 82.36 | 85.47 | 9.95 | 82.34 | 87.01 | 9.69 | 87.36 | 92.58 | 10.59 | *1.00* | *0.88* | *0,89* |
|  | AIE | 113.24 | 116.35 | 24.05 | 52.43 | 57.09 | 4.12 | 55.50 | 60.72 | 4.26 | *5.66* | *5.30* | *0,94* |
|  | PD | 92.16 | 95.27 | 13.17 | 94.15 | 98.82 | 13.58 | 97.89 | 103.11 | 14.31 | *0.94* | *0.86* | *0,92* |
|  | ODG | 102.42 | 105.53 | 17.65 | 104.44 | 109.11 | 18.22 | 107.41 | 112.63 | 18.79 | *0.94* | *0.88* | *0,94* |
|  | PD | 88.53 | 91.64 | 11.87 | 86.45 | 91.12 | 10.89 | 89.48 | 94.70 | 11.25 | *1.06* | *0.99* | *0,94* |
| **mean** | | **88,37** | **91.48** | **12.82** | **81.20** | **85.87** | **10.24** | **85.21** | **90.43** | **10.87** | **1.21** | **1.11** | **0.91** |
| MTL | MSA-C | 87.16 | 90.28 | 11.42 | 75.82 | 80.49 | 8.04 | 92.45 | 97.67 | 12.25 | *1.38* | *0.88* | *0,64* |
|  | MSA-P | 66.34 | 69.45 | 6.30 | 49.87 | 54.54 | 3.83 | 71.44 | 76.66 | 6.72 | *1.59* | *0.88* | *0,55* |
|  | AD | 54.41 | 57.52 | 4.48 | 50.68 | 55.34 | 3.92 | 53.69 | 58.91 | 4.05 | *1.11* | *1.04* | *0,94* |
|  | AD | 81.78 | 84.89 | 9.79 | 59.47 | 64.13 | 5.04 | 86.85 | 92.07 | 10.44 | *1.88* | *0.88* | *0,47* |
|  | AIE | 127.82 | 130.93 | 36.47 | 31.59 | 36.26 | 2.27 | 34.65 | 39.87 | 2.35 | *15.56* | *14.58* | *0,94* |
|  | PD | 77.01 | 80.12 | 8.54 | 78.18 | 82.84 | 8.60 | 82.44 | 87.66 | 9.20 | *0.96* | *0.87* | *0,91* |
|  | ODG | 76.47 | 79.58 | 8.41 | 77.41 | 82.08 | 8.41 | 81.47 | 86.69 | 8.95 | *0.97* | *0.88* | *0,91* |
|  | PD | 89.20 | 92.31 | 12.10 | 67.67 | 72.33 | 6.37 | 94.24 | 99.46 | 12.90 | *1.84* | *0.88* | *0,48* |
| **mean** | | **82,52** | **85.64** | **12.19** | **61.34** | **66.00** | **5.81** | **74.65** | **79.87** | **8.36** | **2.03*** | **1.37** | **0.67** |
| Putamen | MSA-C | 83.60 | 86.71 | 10.31 | 65.43 | 70.09 | 5.97 | 88.87 | 94.09 | 11.06 | *1.67* | *0.88* | *0,52* |
|  | MSA-P | 81.06 | 84.17 | 9.59 | 32.06 | 36.72 | 2.30 | 35.09 | 40.31 | 2.38 | *4.04* | *3.79* | *0,94* |
|  | AD | 69.68 | 72.79 | 6.93 | 71.68 | 76.35 | 7.14 | 74.68 | 79.90 | 7.37 | *0.94* | *0.88* | *0,94* |
|  | AD | 74.08 | 77.19 | 7.85 | 64.71 | 69.38 | 5.85 | 79.44 | 84.66 | 8.45 | *1.30* | *0.87* | *0,67* |
|  | AIE | 124.34 | 127.45 | 33.02 | 42.48 | 47.15 | 3.10 | 45.54 | 50.76 | 3.21 | *10.32* | *9.67* | *0,94* |
|  | PD | 78.27 | 81.38 | 8.85 | 79.37 | 84.03 | 8.90 | 83.74 | 88.96 | 9.55 | *0.96* | *0.87* | *0,90* |
|  | ODG | 91.01 | 94.12 | 12.74 | 93.01 | 97.67 | 13.14 | 96.01 | 101.23 | 13.56 | *0.94* | *0.88* | *0,94* |
|  | PD | 91.56 | 94.67 | 12.94 | 70.70 | 75.37 | 6.95 | 73.73 | 78.95 | 7.18 | *1.81* | *1.69* | *0,94* |
| **mean** | | **86,70** | **89.81** | **12.78** | **64.93** | **69.60** | **6.67** | **72.14** | **77.36** | **7.84** | **1.86*** | **1.53** | **0.82** |

**Table S1** Kinetic modelling overview in the human cohort**.** A superior fit (*) of the 2TC3k compared to the 1TC2k model was found in three target regions due to high values in the autoimmune encephalitis (AIE) patient. F-Tests indicate that the more complex 2TC4k model does not lead to a further significant reduction in the variation of residuals. AIC = Akaike Information Criterion; SC = Schwartz Information Criterion; *χ²* = Sum of Squares of the weighted residuals divided by the degrees of freedom; AD = Alzheimer’s disease continuum; PD = Parkinson’s disease; MSA = multiple systems atrophy; ODG = oligodendroglioma

| ***VOI*** | ***Dx*** | ***VT 1TC2k*** | ***VT Logan*** |
| --- | --- | --- | --- |
| Brainstem | AD (66y, f) | 1.28 ± 0.30 | 1.27 ± 0.30 |
|  | AD (69y, m) | 2.27 ± 0.55 | 2.23 ± 0.53 |
|  | AIE (53y, m) | 2.90 ± 0.72 | 2.85 ± 0.71 |
|  | PD (56y, f) | 1.79 ± 0.37 | 1.76 ± 0.36 |
|  | PD (55y, f) | 1.71 ± 0.39 | 1.67 ± 0.38 |
|  | MSA-C (56y, m) | 2.11 ± 0.66 | 2.22 ± 0.49 |
|  | MSA-P (65y, m) | 2.65 ± 0.77 | 2.47 ± 0.73 |
|  | ODG (60y, f) | 1.17 ± 0.25 | 1.16 ± 0.25 |
| CBW | AD (66y, f) | 1.20 ± 0.20 | 1.19 ± 0.19 |
|  | AD (69y, m) | 1.99 ± 0.31 | 2.00 ± 0.31 |
|  | AIE (53y, m) | 2.67 ± 0.88 | 2.64 ± 0.86 |
|  | PD (56y, f) | 1.57 ± 0.15 | 1.55 ± 0.14 |
|  | PD (55y, f) | 1.54 ± 0.17 | 1.53 ± 0.17 |
|  | MSA-C (56y, m) | 2.11 ± 0.36 | 2.18 ± 0.21 |
|  | MSA-P (65y, m) | 2.64 ± 0.52 | 2.40 ± 0.46 |
|  | ODG (60y, f) | 1.04 ± 0.12 | 1.03 ± 0.12 |
| Composite Cortical | AD (66y, f) | 1.07 ± 0.19 | 1.05 ± 0.19 |
|  | AD (69y, m) | 1.94 ± 0.34 | 1.88 ± 0.32 |
|  | AIE (53y, m) | 1.96 ± 0.38 | 1.93 ± 0.37 |
|  | PD (56y, f) | 1.57 ± 0.26 | 1.51 ± 0.26 |
|  | PD (55y, f) | 1.64 ± 0.27 | 1.59 ± 0.27 |
|  | MSA-C (56y, m) | 1.90 ± 0.40 | 1.87 ± 0.27 |
|  | MSA-P (65y, m) | 2.37 ± 0.45 | 2.10 ± 0.42 |
|  | ODG (60y, f) | 1.04 ± 0.16 | 1.01 ± 0.16 |
| MTL | AD (66y, f) | 1.26 ± 0.22 | 1.22 ± 0.21 |
|  | AD (69y, m) | 2.10 ± 0.41 | 2.00 ± 0.39 |
|  | AIE (53y, m) | 2.18 ± 0.47 | 2.13 ± 0.45 |
|  | PD (56y, f) | 1.72 ± 0.24 | 1.65 ± 0.22 |
|  | PD (55y, f) | 1.72 ± 0.31 | 1.63 ± 0.29 |
|  | MSA-C (56y, m) | 2.01 ± 0.47 | 1.97 ± 0.35 |
|  | MSA-P (65y, m) | 2.58 ± 0.56 | 2.28 ± 0.51 |
|  | ODG (60y, f) | 1.16 ± 0.17 | 1.13 ± 0.16 |
| Putamen | AD (66y, f) | 1.57 ± 0.21 | 1.56 ± 0.20 |
|  | AD (69y, m) | 2.71 ± 0.32 | 2.62 ± 0.28 |
|  | AIE (53y, m) | 2.76 ± 0.45 | 2.72 ± 0.43 |
|  | PD (56y, f) | 2.14 ± 0.40 | 2.07 ± 0.35 |
|  | PD (55y, f) | 2.30 ± 0.39 | 2.20 ± 0.35 |
|  | MSA-C (56y, m) | 2.84 ± 0.39 | 2.72 ± 0.34 |
|  | MSA-P (65y, m) | 3.82 ± 0.49 | 3.35 ± 0.48 |
|  | ODG (60y, f) | 1.43 ± 0.24 | 1.39 ± 0.22 |

**Table S2** Quantitative comparison of Volumes of distribution (V_T_) generated on the basis of a 1TC2k compartmental and a Logan Plot. Intraindividual comparison reveals similar values across all target regions for both quantification methods. AD = Alzheimer’s disease continuum; PD = Parkinson’s disease; MSA = multiple systems atrophy; AIE = autoimmune encephalitis; ODG = oligodendroglioma

**Figures**


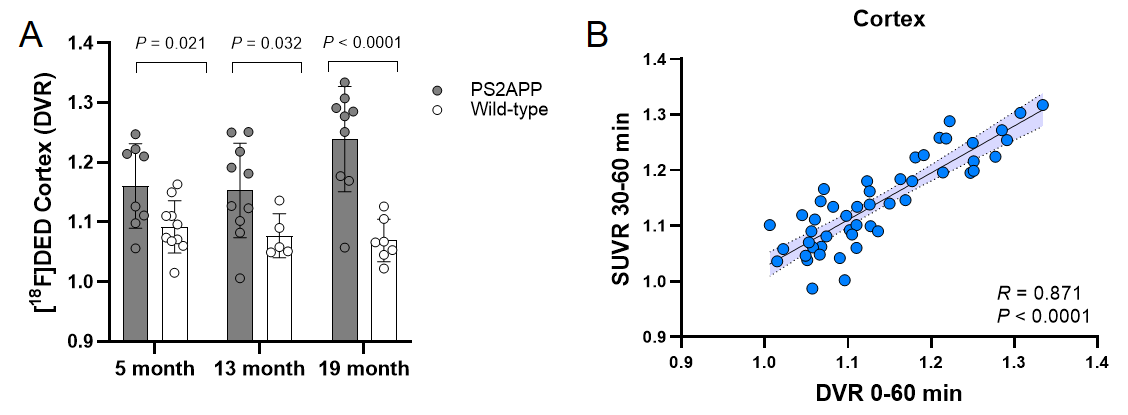


**Figure S1** – (A) Mean (± SD) distribution volume ratios (DVRs) of [^18^F]F-DED PET for PS2APP animals at different ages compared to age-matched wild-type animals for the target region cortex. Significant differences between genotypes per time-point are indicated. (B) Correlation of [^18^F]F-DED DVRs calculated from 60-min dynamic small-animal PET recordings with corresponding 30-60 min SUVR (reference region cerebellum). 95% confidence intervals are represented by dotted lines.


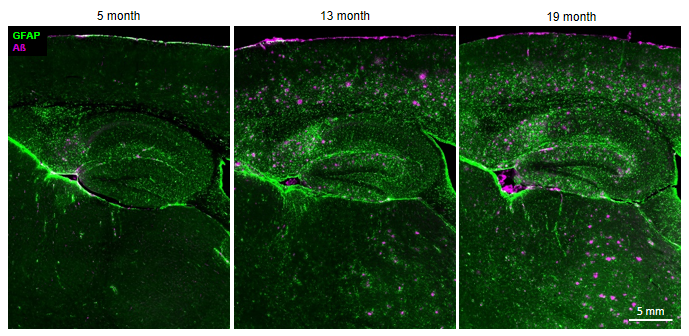


**Figure S2** Sagittal plane showing the cortex, hippocampus and thalamus stained against GFAP for astrocytes and Aβ (NAB228) for Aβ plaques in PS2APP mice at 5, 13 and 19 month of age. Plaques start to from in the subiculum at 5 month of age and spread to the cortex and thalamus. The plaque load is accompanied by astrogliosis which also starts 5 month and increases in an age related manner.

**
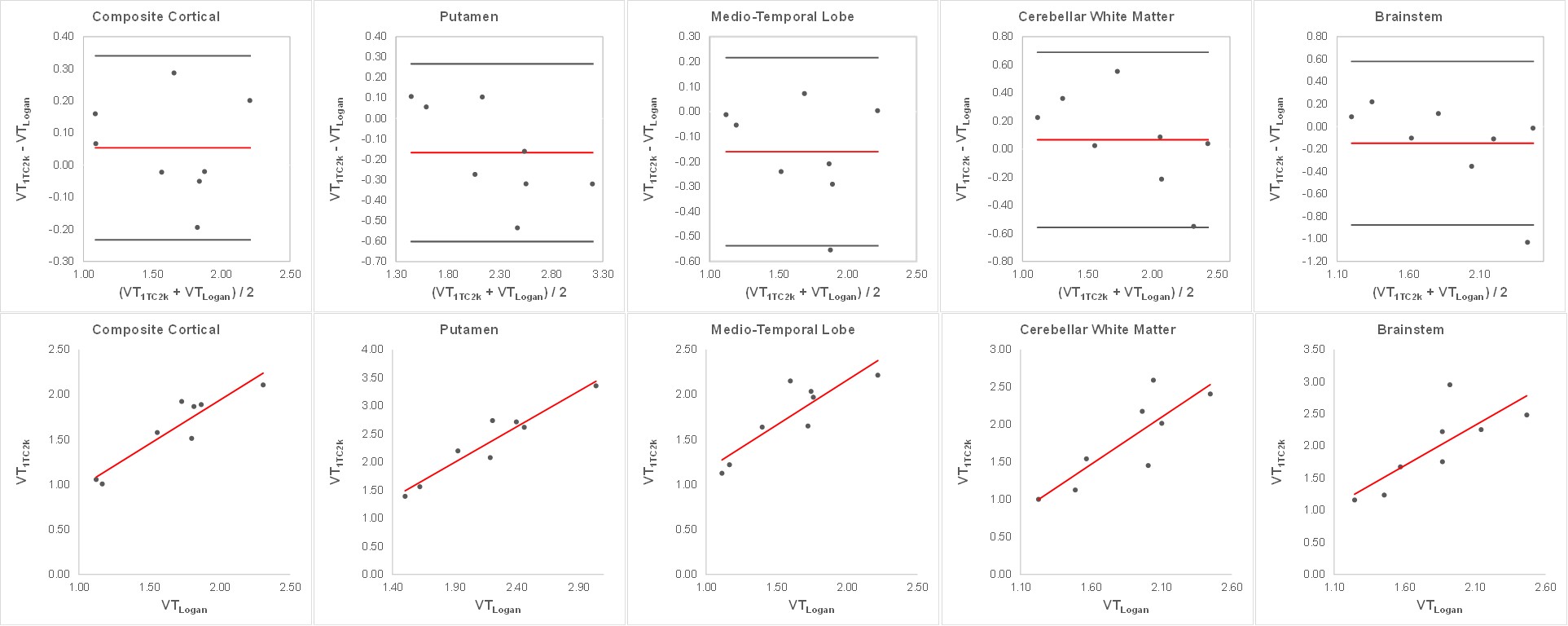
**

**Figure S3** Bland-Altman Plots (first row) comparing V_T_ values based on compartmental and Logan Plot analyses. The red line corresponds to the mean difference of V_T_ values, the black lines indicate the limits of agreement (Mean ± 1.96 * SD). Correlational analyses (second row) reveal high correlations between V_T_ values based on the 1TC2k and those based on Logan Plot.


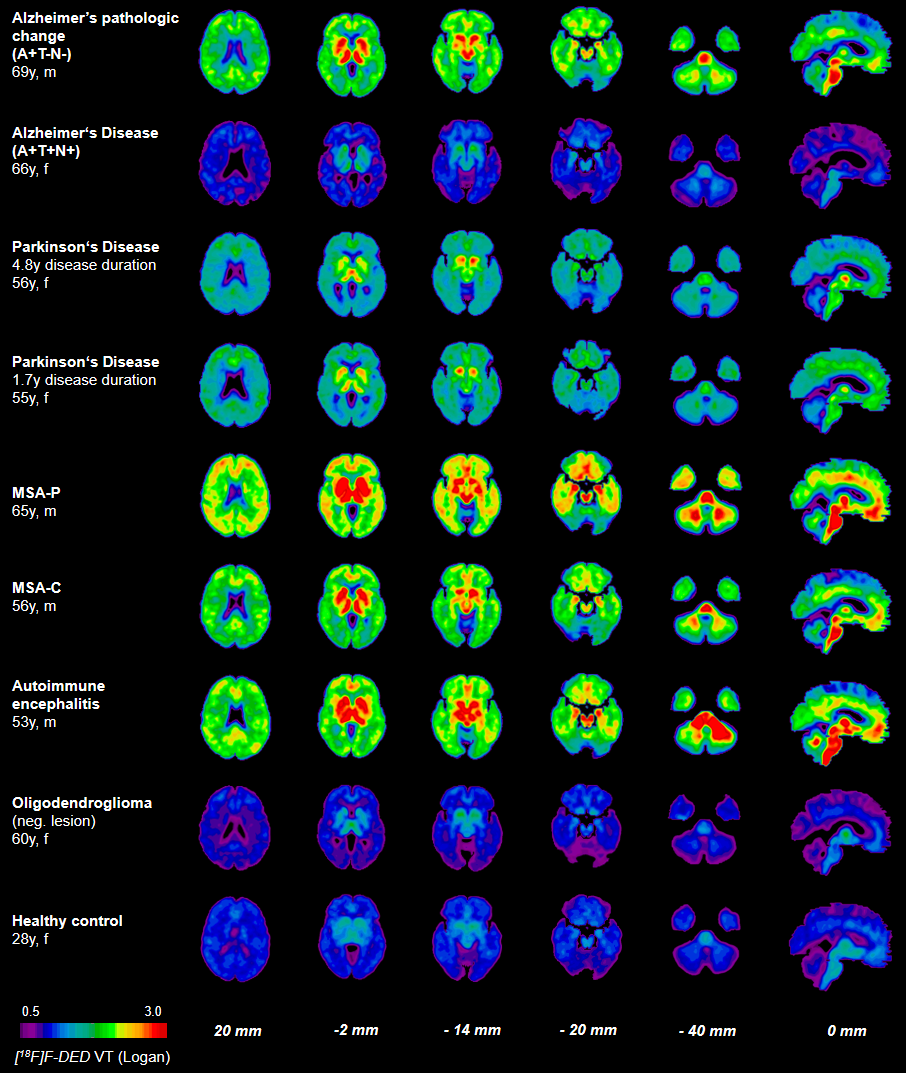


**Figure S4** Axial and sagittal planes show [^18^F]F-DED Volumes of distribution (V_T_) based on a Logan Plot at levels of neocortical regions, basal ganglia, hippocampus, cerebellum (all coronal) and brainstem (sagittal). The lesions of patients with autoimmune encephalitis and oligodendroglioma are indicated with white arrows.


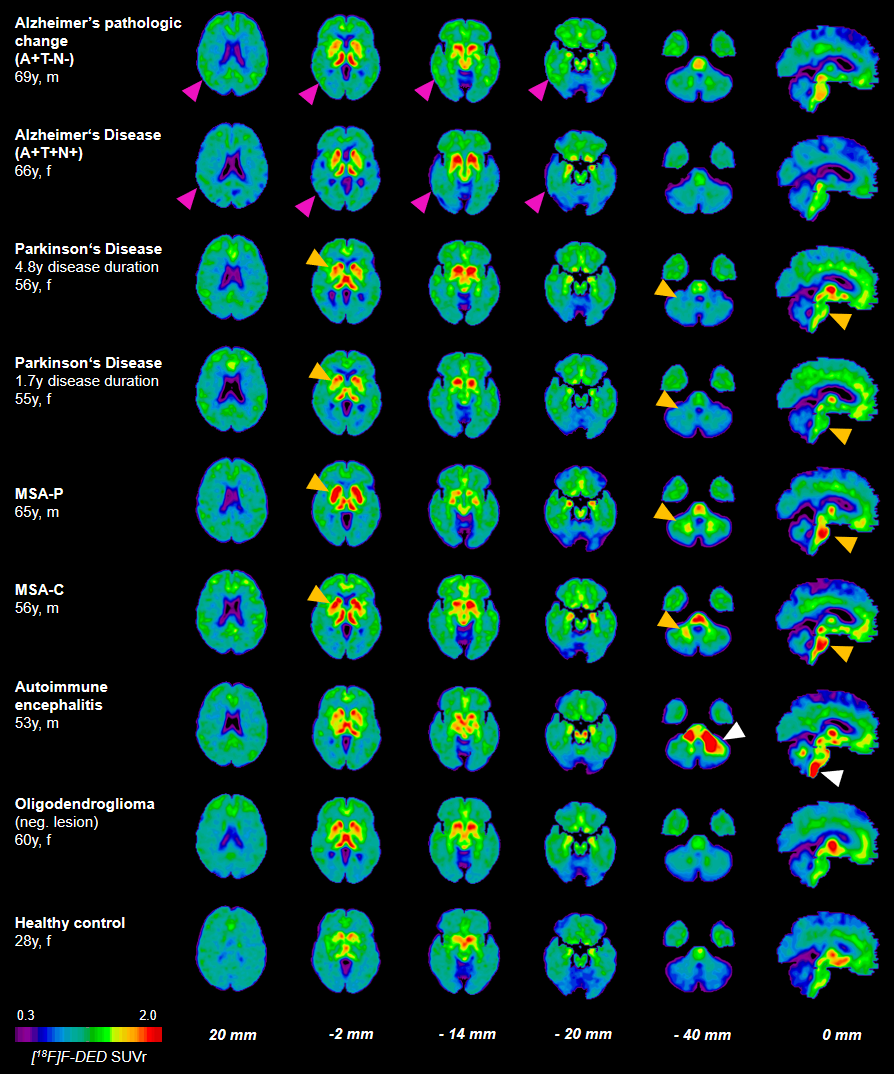


**Figure S5** Axial and sagittal planes show [^18^F]F-DED standardized uptake value ratios (SUVr) based on a parietal white matter reference region and a 30-60 min time frame at levels of neocortical regions, basal ganglia, hippocampus, cerebellum (all coronal) and brainstem (sagittal). Scaling is optimized to evaluate signal changes in the basal ganglia and brainstem regions. Parietal and temporal Alzheimer’s disease signature regions are indicated with pink arrows. Regions of interest in patients with Parkinson’s disease and multiple systems atrophy (MSA) are indicated with orange arrows. The lesions of the patient with autoimmune encephalitis are indicated with white arrows.


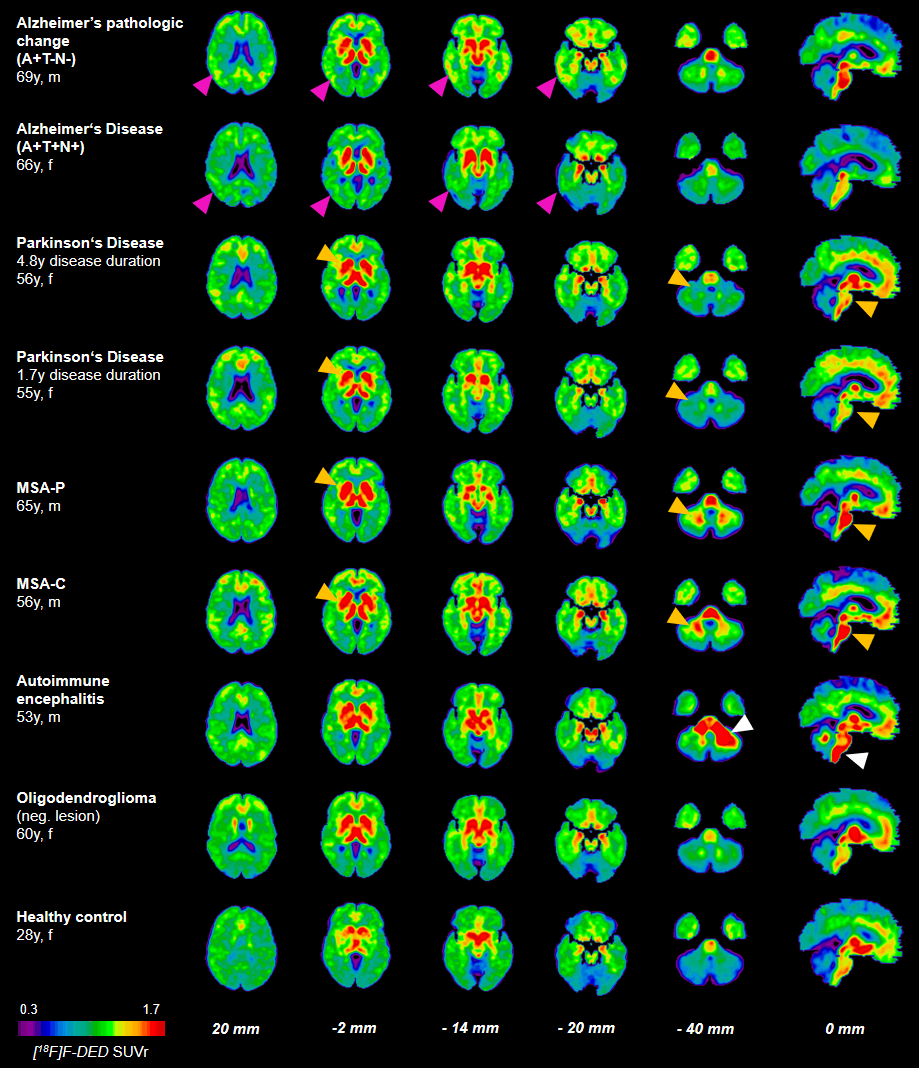


**Figure S6** Axial and sagittal planes show [^18^F]F-DED standardized uptake value ratios (SUVr) based on a parietal white matter reference region and a 30-60 min time frame at levels of neocortical regions, basal ganglia, hippocampus, cerebellum (all coronal) and brainstem (sagittal). Scaling is optimized to evaluate signal changes in the cortical regions. Parietal and temporal Alzheimer’s disease signature regions are indicated with pink arrows. Regions of interest in patients with Parkinson’s disease and multiple systems atrophy (MSA) are indicated with orange arrows. The lesions of the patient with autoimmune encephalitis are indicated with white arrows.


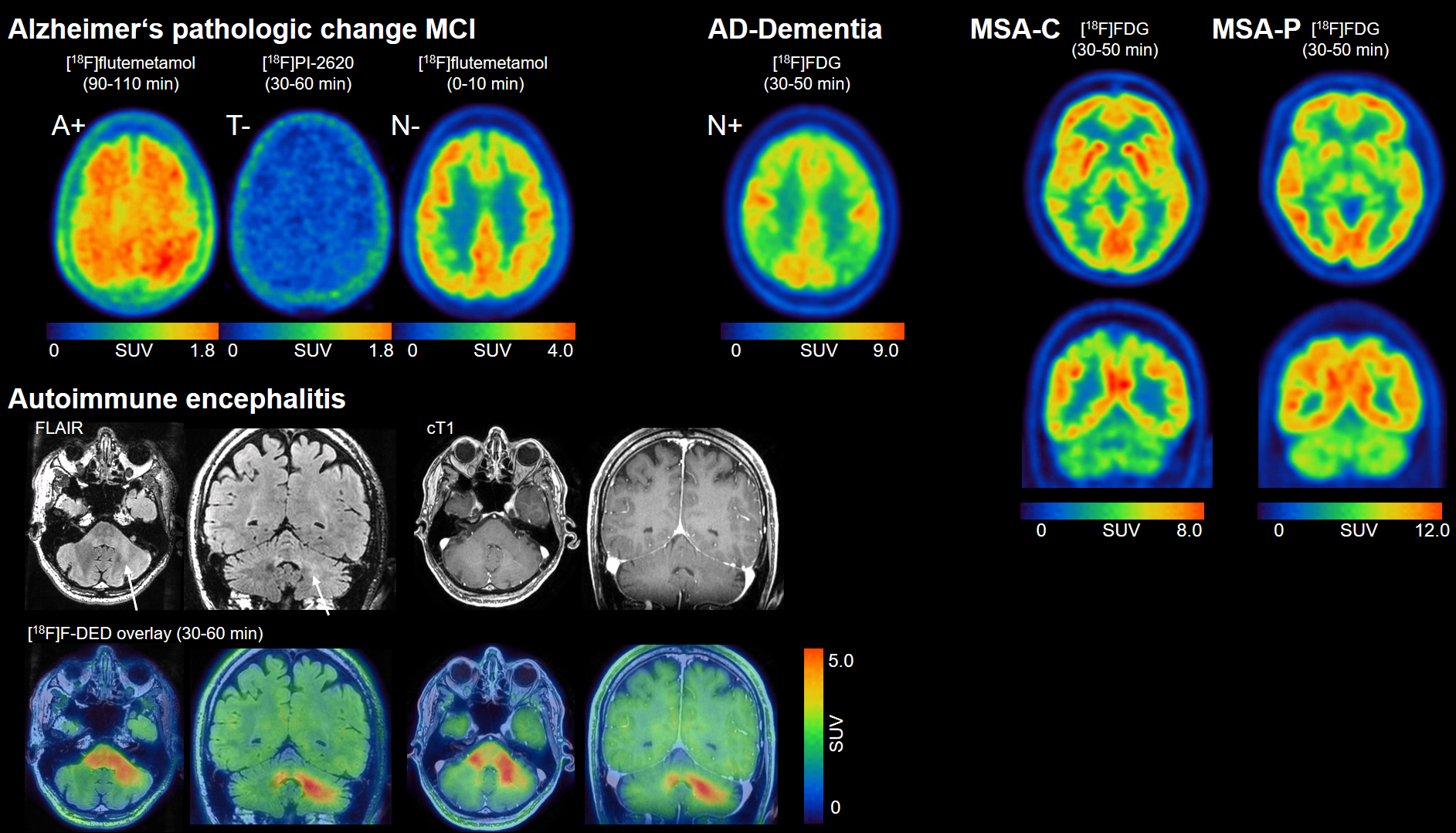


**Figure S7** Additional imaging characteristics of investigated patients of the Alzheimer’s disease (AD) continuum, multiple systems atrophy (MSA) and autoimmune encephalitis as assessed by β-amyloid-PET, tau-PET, FDG-PET and MRI


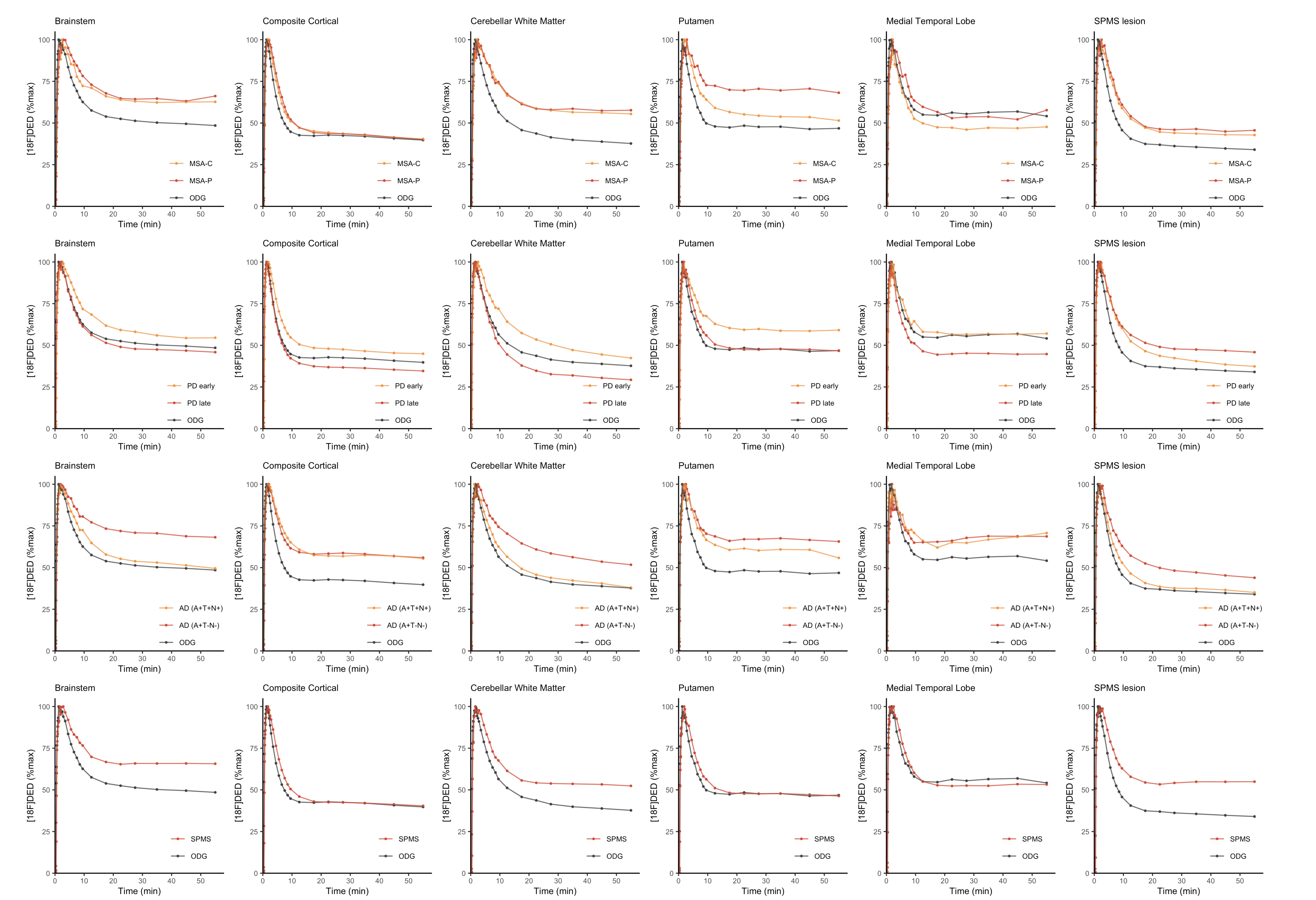


**Figure S8** - [^18^F]F-DED time-activity-curves (TACs) of patients with neurodegenerative diseases and multiple sclerosis in contrast to a patient with MAO-B negative oligodendroglioma. All TACs are scaled to the maximum SUV to allow direct comparison of the wash-out phase. MSA-P = Multiple systems atrophy - parkinsonian subtype; MSA-C = Multiple systems atrophy - cerebellar subtype; ODG = Oligodendroglioma; PD = Parkinson’s disease; AD = Alzheimer’s disease (A = amyloid-β; T = tau; N = neurodegeneration); SUV = standard-uptake-value; min = minutes.


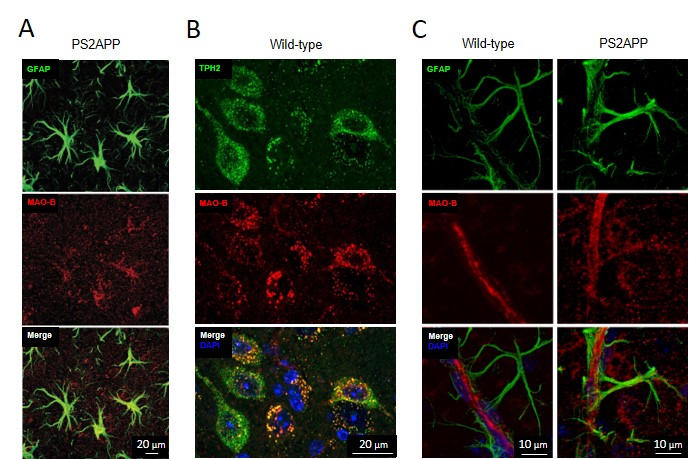


**Figure S9** (**A**) MAO-B expression in GFAP-(+) astrocytes, (**B**) TPH2-(+) serotonergic neurons of the raphe nucleus and (**C**) in endothelia cells of blood vessels of wild-type and PS2APP mice.
